# Genotype–phenotype correlation in *PRKN-*associated Parkinson’s disease

**DOI:** 10.1038/s41531-024-00677-3

**Published:** 2024-03-29

**Authors:** Poornima Jayadev Menon, Sara Sambin, Baptiste Criniere-Boizet, Thomas Courtin, Christelle Tesson, Fanny Casse, Melanie Ferrien, Louise-Laure Mariani, Stephanie Carvalho, Francois-Xavier Lejeune, Sana Rebbah, Gaspard Martet, Marion Houot, Aymeric Lanore, Graziella Mangone, Emmanuel Roze, Marie Vidailhet, Jan Aasly, Ziv Gan Or, Eric Yu, Yves Dauvilliers, Alexander Zimprich, Volker Tomantschger, Walter Pirker, Ignacio Álvarez, Pau Pastor, Alessio Di Fonzo, Kailash P. Bhatia, Francesca Magrinelli, Henry Houlden, Raquel Real, Andrea Quattrone, Patricia Limousin, Prasad Korlipara, Thomas Foltynie, Donald Grosset, Nigel Williams, Derek Narendra, Hsin-Pin Lin, Carna Jovanovic, Marina Svetel, Timothy Lynch, Amy Gallagher, Wim Vandenberghe, Thomas Gasser, Kathrin Brockmann, Huw R. Morris, Max Borsche, Christine Klein, Olga Corti, Alexis Brice, Suzanne Lesage, Jean Christophe Corvol, French Parkinson disease Genetics Study Group (PDG)

**Affiliations:** 1 Sorbonne Université, Institut du Cerveau – Paris Brain Institute – ICM, Inserm, CNRS, Paris, France; 2 Assistance Publique Hôpitaux de Paris, Department of Neurology, CIC Neurosciences, Hôpital Pitié-Salpêtrière, Paris, France; 3 School of Postgraduate Studies, Royal College of Surgeons in Ireland, Dublin, Ireland; 4 Assistance Publique Hôpitaux de Paris, Department of Genetics, Hôpital Pitié-Salpêtrière, Paris, France; 5 Centre of Excellence of Neurodegenerative Disease (CoEN), AP-HP, Pitié-Salpêtrière Hospital, Paris, France; 6 Institute of Memory and Alzheimer’s Disease (IM2A), Department of Neurology, AP-HP, Pitié-Salpêtrière Hospital, Paris, France; 7 Department of Neurology, Movement Disorder Division, Rush University Medical Center, 1725 W. Harrison Street, Chicago, IL USA; 8 Department of Neurology, Norwegian University of Science and Technology, Trondheim, Norway; 9 The Neuro (Montreal Neurological Institute-Hospital), McGill University, Montreal, QC Canada; 10 Department of Neurology and Neurosurgery, McGill University, Montreal, QC Canada; 11 Department of Human Genetics, McGill University, Montreal, QC Canada; 12 Department of Neurology, Gui-de-Chauliac Hospital, CHU Montpellier, University of Montpellier, Institute for Neurosciences of Montpellier (INM), INSERM, Montpellier, France; 13 Department of Neurology, University Hospital Vienna, Vienna, Austria; 14 Gailtal-Klinik Hermagor, Hermagor, Austria; 15 Department of Neurology, Ottakring Clinic, Vienna, Austria; 16 Department of Neurology, Hospital Universitari Mutua de Terrassa, and Fundació per a la Recerca Biomèdica i Social Mútua de Terrassa, Terrassa, Barcelona Spain; 17 Unit of Neurodegenerative diseases, Department of Neurology, University Hospital Germans Trias i Pujol and The Germans Trias i Pujol Research Institute (IGTP) Badalona, Barcelona, Spain; 18 Fondazione IRCCS Ca’ Granda Ospedale Maggiore Policlinico, Milan, Italy; 19 Department of Clinical and Movement Neurosciences, UCL Queen Square Institute of Neurology, University College London, London, UK; 20 Department of Neuromuscular Diseases, UCL Queen Square Institute of Neurology, London, UK; 21 UCL Movement Disorders Centre, University College London, London, UK; 22 Aligning Science Across Parkinson’s (ASAP) Collaborative Research Network, Chevy Chase, MD 20815 USA; 23 Institute of Neurology, Department of Medical and Surgical Sciences, Magna Graecia University of Catanzaro, Catanzaro, Italy; 24 Institute of Neurological Sciences, University of Glasgow, Glasgow, UK; 25 Department of Psychological Medicine and Neurology, Cardiff University, Cardiff, UK; 26 Inherited Disorders Unit, Neurogenetics Branch, Division of Intramural Research, National Institute of Neurological Disorders and Stroke, National Institutes of Health, Bethesda, MD USA; 27 University Clinical Center of Serbia, Neurology Clinic, Belgrade, Serbia; 28 The Dublin Neurological Institute at the Mater Misericordiae University Hospital, Dublin Ireland and University College Dublin, Dublin, Ireland; 29 Department of Neurology, University Hospitals Leuven; Department of Neurosciences, KU Leuven; Leuven Brain Institute, Leuven, Belgium; 30 Department of Neurodegenerative Diseases, Hertie Institute for Clinical Brain Research, University of Tübingen, Tübingen, Germany; 31 DZNE, German Center for Neurodegenerative Diseases, Tübingen, Germany; 32 Institute of Neurogenetics, University of Lübeck, Lübeck, Germany

**Keywords:** Parkinson's disease, Prognostic markers, Parkinson's disease, Basal ganglia, Mutation

## Abstract

Bi-allelic pathogenic variants in *PRKN* are the most common cause of autosomal recessive Parkinson’s disease (PD). 647 patients with *PRKN*-PD were included in this international study. The pathogenic variants present were characterised and investigated for their effect on phenotype. Clinical features and progression of *PRKN*-PD was also assessed. Among 133 variants in index cases (*n* = 582), there were 58 (43.6%) structural variants, 34 (25.6%) missense, 20 (15%) frameshift, 10 splice site (7.5%%), 9 (6.8%) nonsense and 2 (1.5%) indels. The most frequent variant overall was an exon 3 deletion (*n* = 145, 12.3%), followed by the p.R275W substitution (*n* = 117, 10%). Exon3, RING0 protein domain and the ubiquitin-like protein domain were mutational hotspots with 31%, 35.4% and 31.7% of index cases presenting mutations in these regions respectively. The presence of a frameshift or structural variant was associated with a 3.4 ± 1.6 years or a 4.7 ± 1.6 years earlier age at onset of *PRKN*-PD respectively (*p* < 0.05). Furthermore, variants located in the N-terminus of the protein, a region enriched with frameshift variants, were associated with an earlier age at onset. The phenotype of *PRKN*-PD was characterised by slow motor progression, preserved cognition, an excellent motor response to levodopa therapy and later development of motor complications compared to early-onset PD. Non-motor symptoms were however common in *PRKN*-PD. Our findings on the relationship between the type of variant in *PRKN* and the phenotype of the disease may have implications for both genetic counselling and the design of precision clinical trials.

## Introduction

Parkinson’s disease (PD) is commonly sporadic, but 10% of patients present a familial form of the disease, with genetic forms resulting from either autosomal dominant or recessive inheritance^1^. Although rare, the description of these genetic forms of the disease has brought important insights into the causal pathophysiological mechanisms of sporadic PD.

Bi-allelic pathogenic variants in the *PRKN* gene are the most common cause of autosomal recessive PD, accounting for between 2.6% and 14.9% of cases of early-onset PD (age at onset ≤50 years) depending on the population^2–5^. The typical presentation of *PRKN*-PD is characterised by an early age at onset, usually before 45, a pure motor disease with an excellent response to dopaminergic therapy, slow progression, and a lack of cognitive decline^6–9^. In accordance with this phenotype, neuropathological features of *PRKN*-PD showed that it is predominantly a ‘pure nigropathy’, with severe loss of dopaminergic neurons in the substantia nigra and minimal Lewy bodies in comparison to idiopathic PD (IPD)^10^. There is typically sparing of the nucleus basalis of Meynert and the cerebral cortex, which is thought to reflect the lack of cognitive involvement in *PRKN*-PD^10^.

There is however an important variability in the phenotype of *PRKN*-PD, both on presentation and during progression. In a large international database, there was a wide distribution of the age at onset, with a median age of 31 years, but a range of 3–81 years^7^. Importantly, despite the slow motor progression, a younger age at onset is thought to be associated with greater accumulation of motor disability in PD^11,12^. Motor complications are frequent in patients with *PRKN*-PD including peak-dose dyskinesia^9^. Finally, the pathology is also characterised by mild involvement of the locus coerulus and the dorsal motor nucleus of the vagus^10^, and non-motor features have also been suggested in certain studies, with one study reporting that 60% of patients had autonomic dysfunction and 56% described behavioural/psychiatric symptoms^9^. The cause of this variability across patients, and its relationship with the different pathogenic variants in the *PRKN* gene remains largely unknown.

*PRKN* contains 12 exons that encode the 465 amino-acid protein, Parkin^13^. Parkin is involved in ubiquitination of substrate proteins and mitochondrial quality control^14–16^. It is thought that impaired mitophagy in *PRKN-*PD patients results in the accumulation of cytotoxic levels of reactive oxygen species detrimental to dopaminergic neurons, however, the specific mechanisms linking Parkin dysfunction and PD is not fully elucidated yet^17,18^. There are up to 140 different pathogenic loss-of-function variants in *PRKN*, including missense, frameshift, nonsense, splice site variants, as well as exon deletions or multiplications^2,19^. The evidence for an effect of specific variant or variant type on phenotype in bi-allelic *PRKN*-PD is currently ambiguous, with only a couple of studies with small sample size having investigated this question^20,21^.

In the present large multi-centre cohort study, we aimed to investigate the effect of specific pathogenic variants or variant type on the phenotype of *PRKN*-PD. Variants were classified depending on their location and their consequences on the gene and the protein. Age at onset, Hoehn and Yahr stage, and Unified Parkinson’s disease Rating Scale (UPDRS) III motor progression were the main clinical endpoints. For the subset of patients for whom longitudinal data were available, we also describe motor and non-motor complications.

## Results

### Clinical characteristics

Data from a total of 647 patients were collected, 253 patients from the Michael J Fox Foundation (MJFF) study, 227 from the Noyaux Gris Centraux (NGC)/NS-Park database, and 167 from the Genotype–Phenotype correlation in *PRKN-*PD (GPiP) participating centres. Individuals in this cohort were from 46 different countries of origin, with most individuals being of Caucasian origin (76.5%). Fifty-nine percent of cases had a family history of *PRKN*-PD (*n* = 563).

The clinical characteristics of the patients at last clinical examination are detailed in Table 1. The cohort had a mean disease duration of 18.2 ± 12.5 (Table 1) years from onset of first motor symptom to time of last clinical assessment. The age at onset of PD ranged from 7 to 71 years, with a mean of 31.4 ± 11.34 years (Supplementary Fig. 1).

**Table 1:** Demographic and clinical characteristics of cohort

Demographic and clinical characteristics	Number
Female : Male (*n* = 632)	308:324
Mean age at onset (years) (SD) (*n* = 615)	31.4 ± 11.34
Distribution of age at onset:	
• Percentage of individuals < 21 years of age	20%
• Percentage of individuals 21 – 49 years of age	74.3%
• Percentage of individuals 50 – 59 years of age	4.2%
• Percentage of individuals > 59 years of age	1.5%
Mean disease duration in years at examination (SD) (*n* = 503)	18.2 ± 12.5
Mean UPDRS part III (on)/108 (SD) (*n* = 354)	20 ± 15
Mean UPDRS part III (off)/108 (SD) (*n* = 60)	33.5 ± 17
Mean Hoehn and Yahr (on)/5 (SD) (*n* = 359)	2.1 ± 0.9
Mean Hoehn and Yahr (off)/5 (SD) (*n* = 69)	2.66 ± 1.2
Mean MMSE/30 (SD) (*n* = 253)	28.4 ± 3.5
Mean LEDD (mg) (SD) (*n* = 252)	500 ± 455
Number of patients with DBS at time of examination (*n* = 137)	23
Mean disease duration at time of DBS (years) (SD) (*n* = 20)	23.7 ± 10.1

There was no difference in the age at onset between the three different groups: NGC, MJFF and GPiP centres (Supplementary Table 1). There were no differences in age at onset, disease duration, Mini-mental state examination (MMSE) scores, or levodopa equivalent daily dose (LEDD) scores between men and women in the total cohort.

### Variant-specific information

The prevalence of variants was assessed in index cases (*n* = 582). We identified 133 different *PRKN* variants in the cohort (Supplementary Table 2), including 20 variants previously not reported (Supplementary Table 3)^22,23^. Ten individuals had 3 independent pathogenic variants in *PRKN*. Four patients had one other variant in a known autosomal recessive PD gene in addition to the two variants in *PRKN*: two patients with one variant in *PINK1*, one patient with one variant in *ATP13A2*, and one patient with one variant in *SYNJ1*.

**Fig. 1:**
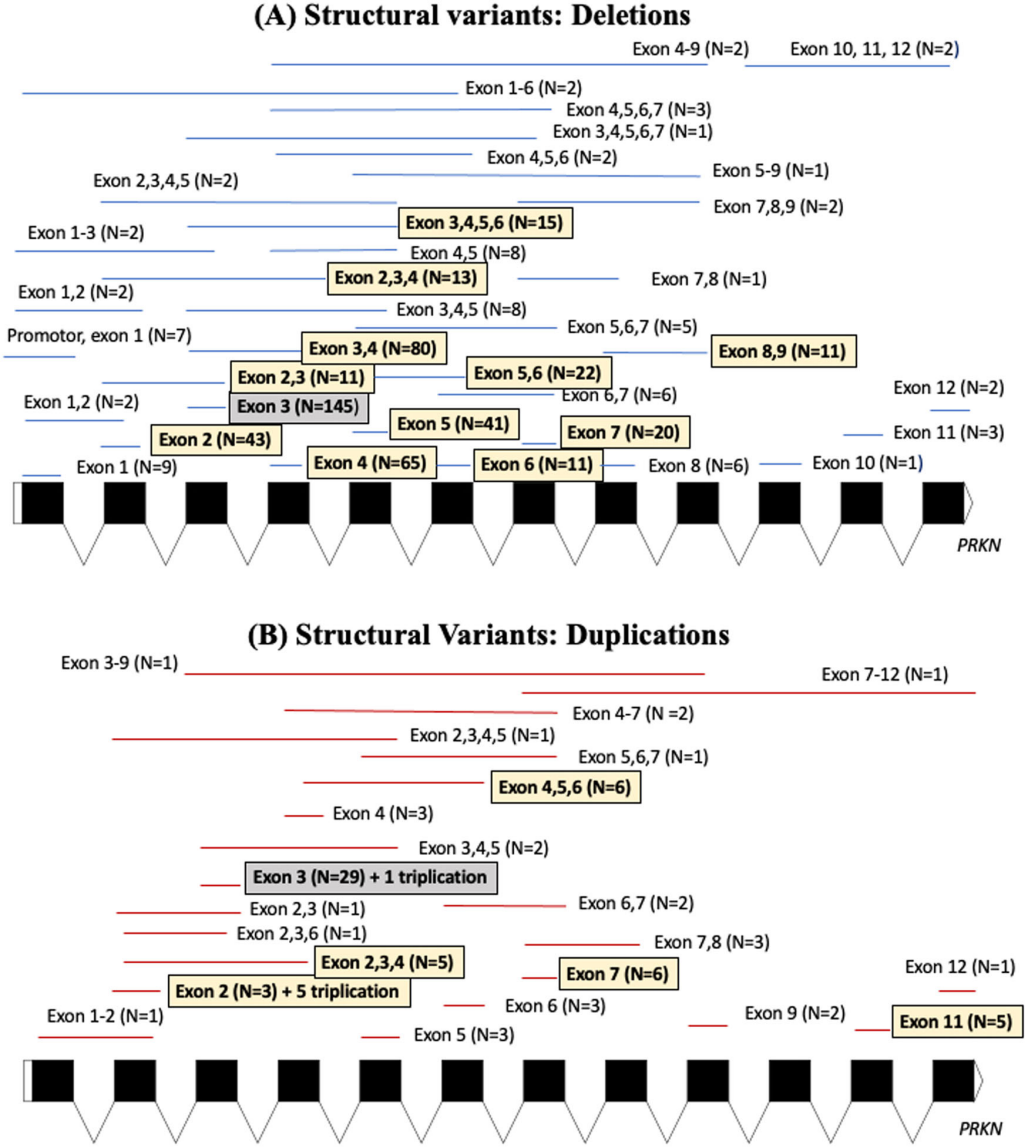
Exonic locations of structural variants identified in the cohort. **A** The exonic locations of deletions identified in the cohort. **B** The exonic locations of duplications identified in the cohort. *N* refers to the number of times these variants were identified in the cohort, with the homozygous presence of these variants being counted as *N* = 2.

**Fig. 2:**
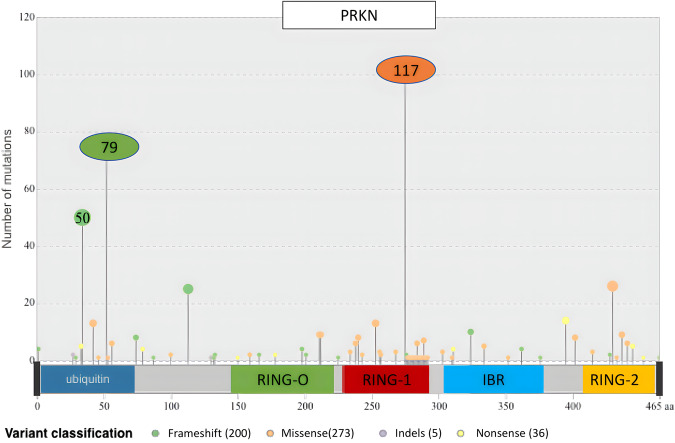
Lollipop plot demonstrating the Parkin protein domain location of single nucleotide variants identified in the cohort. (The size of the lollipop corresponds to the frequency at which variants were identified at these loci).

Supplementary Table 4 details the molecular features of the variants in the index cases including the type of variants, exonic location and protein domains affected. Figure 1 depicts the exonic deletions and duplications present in the cohort. Figure 2 shows the location of the single nucleotide variants in reference to the Parkin protein and the frequency at which these variants were encountered in the cohort. Virtually all (98%) single nucleotide variants present in the cohort had Combined Annotation Dependent Depletion (CADD) scores greater than 20. (Ten splice site variants were also present in the cohort but have not been shown in the figures).

The most common combination of pathogenic *PRKN* variants in index cases were two structural (copy number) variants (39.2%), followed by a missense and a structural variant (17.7%), a structural and a frameshift variant (10.3%), two missense variants (9.6%), two frameshift variants (8.4%), and then a missense and a frameshift variant (6.2%) (Supplementary Table 4). Bi-allelic variants involving nonsense, splice site and indels were less common. The most frequent variant in index cases was an exon 3 deletion (*n* = 145, 12.3%), followed by the p.R275W substitution (*n* = 117, 10%), deletion of exon 3,4 (*n* = 80, 6.8%) and p.N52Mfs*29 frameshift variant (*n* = 79, 6.7%), respectively (Supplementary Table 2). The most frequent exon involved in index cases by the different types of variants was exon 3 (31%), while the most frequent protein domain involved was the RING0 domain (35.4%) followed by the ubiquitin-like domain (31.7%).

### Association between features of *PRKN* variants and age at onset

The association analysis was undertaken in index cases and relatives with *PRKN* PD (Supplementary Fig. 2).

**Fig. 3:**
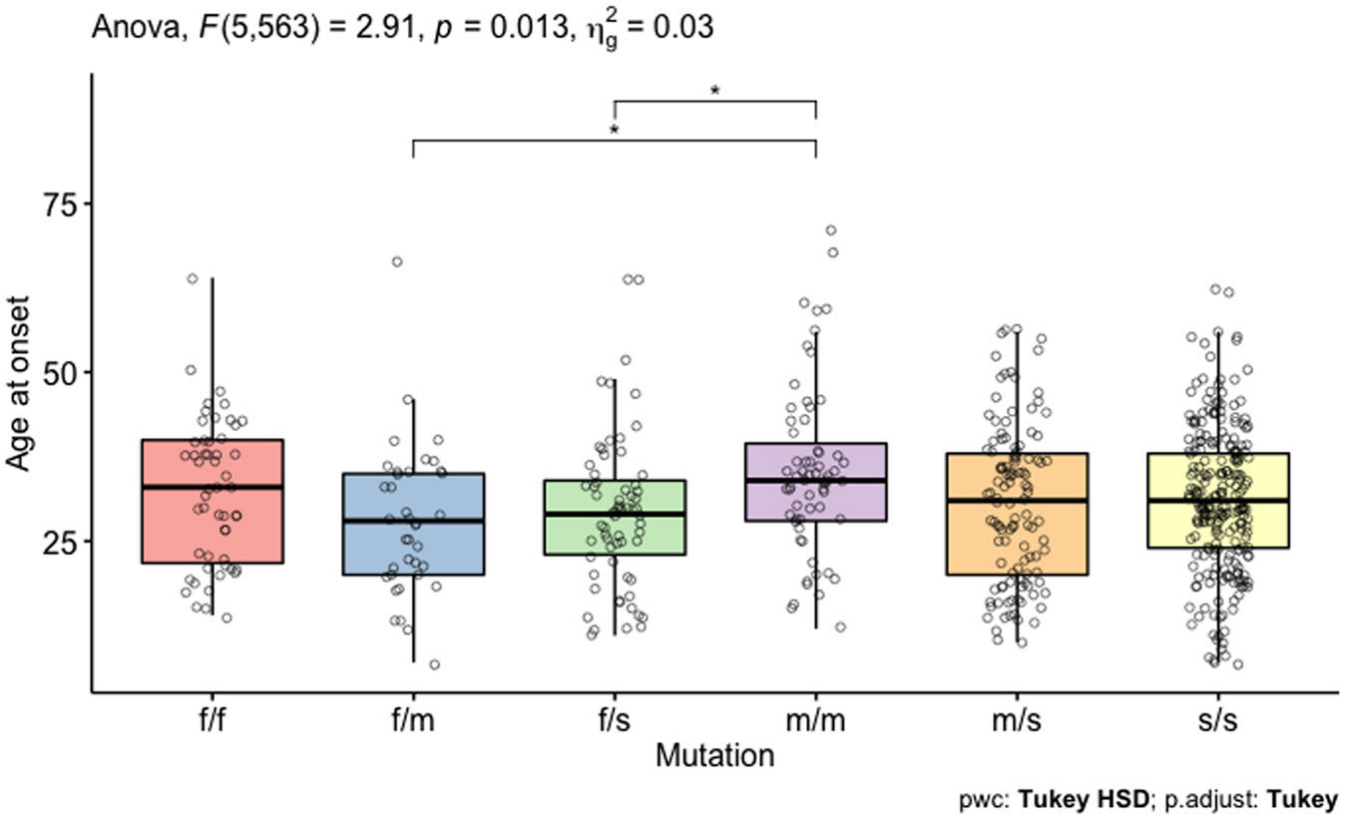
Boxplot demonstrating the average age at onset of *PRKN-*PD based on the type of variant. (f/f = frameshift/frameshift, f/m = frameshift/ missense, f/s = frameshift/ structural, m/m = missense/missense, m/s = missense/structural, s/s = structural/structural).

Age at onset of *PRKN*-PD was influenced by the type of *PRKN* variant (*F*_5,563_ = 2.91, *p* = 0.013). Patients with two missense variants (m/m, *n* = 63) had the oldest age at onset of PD at 35.4 ± 12.5 years in comparison with 27.9 ± 11.1 years (*n* = 39) for those with a frameshift and a missense variant and 29.4 ± 11.3 years for those with a frameshift and a structural variant (*n* = 66) (Fig. 3). The age at onset varied not only based on the type of variant, but also by considering the sex of the individual, along with the type of variant. Women with two missense variants (*n* = 25) had a 7.5 ± 2.9 years later age at onset compared to men with 2 missense variants (*n* = 38) (*p* < 0.05) (Supplementary Fig. 3). Adjusting for the effect of sex, patients who possessed at least one frameshift variant had a 3.4 ± 1.6 years earlier age at onset (*p* = 0.04), while those with at least one structural variant had a 4.7 ± 1.6 years earlier age at onset (*p* = 0.003) compared to a mean age of 35 years in a female who did not have a missense, frameshift or structural variant.

When considering the effect of total number of exons altered, duplicated or absent due to each of the two variants, the age at onset was influenced by the number of exons involved, with involvement of each additional one exon reducing the age by 0.14 ± 0.06 years, starting from an estimated mean age at onset of 33 years (*p* = 0.03).

**Fig. 4:**
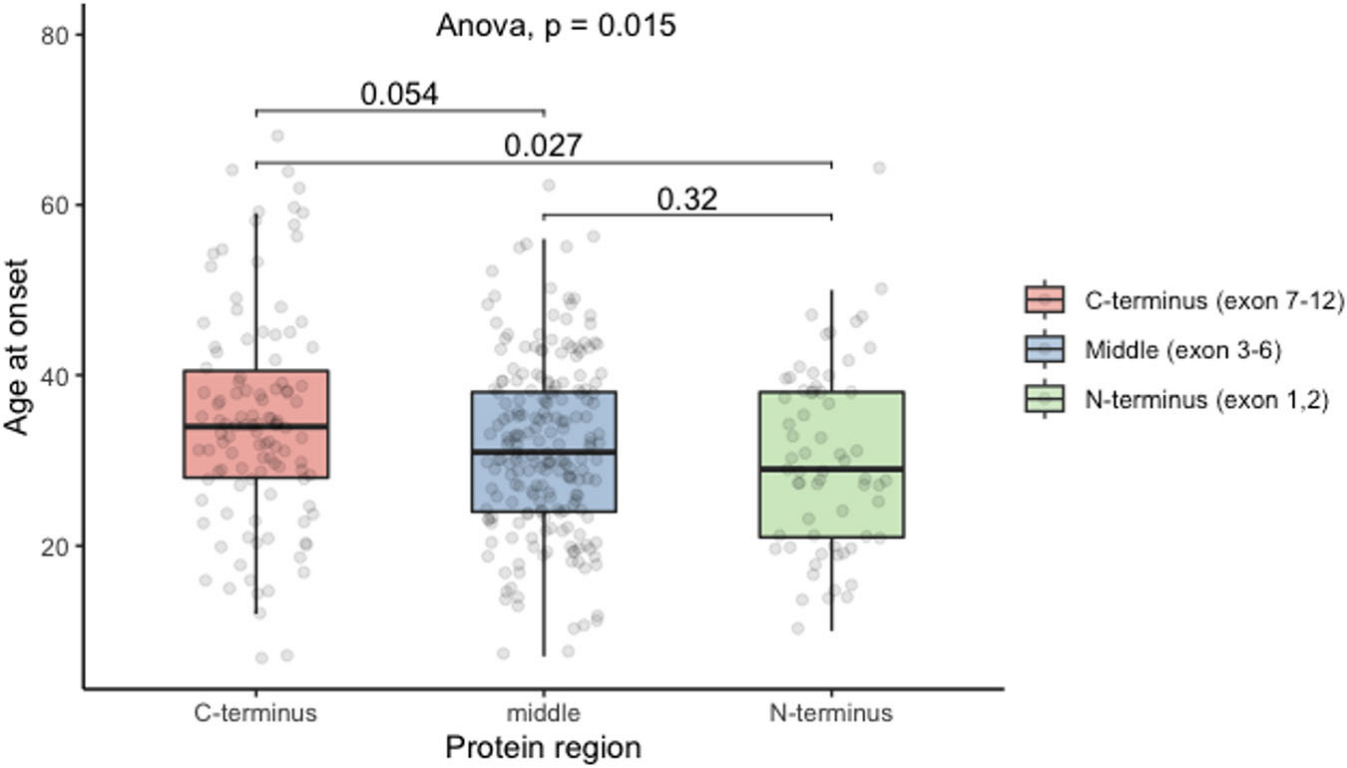
Boxplot demonstrating the average age at onset of *PRKN-PD* based on the Parkin protein terminus location of variants. (C-terminus = exon 7–12, Middle = exon 3–6, N-terminus = exon 1–2).

Amongst patients with the two variants located within the same region of the protein, age at onset was significantly different based on whether these variants were located in the C-terminus (exons 7–12), middle (exon 3–6) or N-terminus (exon 1–2) of the Parkin protein (Fig. 4, *n* = 379, Analysis of Variance (ANOVA) *p* = 0.015). Compared to patients with the 2 variants located at the C-terminus (*n* = 111) who had a mean age at onset of 34.8 ± 12.7 years, individuals who had two variants located at the N-terminus of the protein (exon 1–2, *n* = 64) had a 4.5 ± 1.7 years earlier age at onset of PD (post-hoc Tukey, *p* = 0.027), while those with the two variants in the middle (exon 3–6, *n* = 204) had a 3.1 ± 1.3 years earlier age at onset (post-hoc Tukey, *p* = 0.054). Age at onset was not significantly different between patients with two variants in the N-terminus and those with the two variants in the middle of the protein (post-hoc Tukey, *p* = 0.32). There were 39 frameshift/frameshift variants in the N-terminus, nine in the middle and three in the C-terminus, 14 structural/structural variants in the N-terminus, 169 in the middle and 32 in the C-terminus and four missense/missense variants in the N-terminus, 5 in the middle and 44 in the C-terminus (*p* < 0.05).

There was no association between age at onset and the protein domain location of the variants (*n* = 76 for 2 ubiquitin-like domains, *n* = 77 for 2 RING0 domains, *n* = 34 for 2 RING1 domains, *n* = 16 for 2 RING2 domains), the amino acid location of missense (*n* = 63) and frameshift variants (*n* = 55) in the protein and CADD scores (*n* = 214). There was no significant difference in age at onset between those who had variants involving exon 3 (*n* = 280) and those with variants involving the other exons (*n* = 367). There were also no significant differences between those who possessed homozygous exon 3 deletions (*n* = 54) in comparison with those who possessed homozygous exon 3 duplications (*n* = 9) or between those who had 2 exonic deletions (*n* = 213) compared to 2 exonic duplications (*n* = 28) within the entire cohort.

Homozygous exon 3 deletions (*n* = 54) and a homozygous N52Mfs*29 variants (*n* = 26) were the most common homozygous genotypes. Interestingly, the distribution of age at onset for these sub-populations with the same genotype ranged from 10 to 49 years (homozygous exon 3 deletion), and 15–64 years (homozygous N52Mfs*29 variant) suggesting that other factors may participate in the variability of the age at onset (Supplementary Fig. 4).

The type of variant, total number of exons affected, or protein terminus location of variants combined with the duration of disease were not associated with UPDRS III (ON) scores or Hoehn and Yahr (ON) scores.

### Motor symptoms

Clinical data on motor and non-motor complications were available for 152 patients (73 men, 79 women) (Supplementary Fig. 5). Insufficient sample size hindered our ability to achieve adequate statistical power to detect meaningful relationship between the type of *PRKN* variant and motor and non-motor complications.

These patients (*n* = 104) had an average delay of 8.3 ± 7.5 years from the first motor symptom to diagnosis as PD. The first motor symptom for most of these patients was tremor (46%), followed by dystonia (32%) (*n* = 96). The age at onset based on the first motor symptom was 27.8 ± 10 years (*n* = 44) for tremor, 28 ± 12 years (*n* = 31) for dystonia, 30.5 ± 10.5 years (*n* = 8) for rigidity/bradykinesia, 31.6 ± 8.5 years (*n* = 5) for postural instability and 36 ± 4 years (*n* = 5) for gait disturbance (*p* = 0.37).

A significant proportion of these individuals had motor complications including motor fluctuations (65%, *n* = 127), dyskinesia (62.5%, *n* = 128), freezing of gait (43%, *n* = 124) and postural instability (53.4%, *n* = 116) at time of last examination.

### Modelling motor progression

**Fig. 5:**
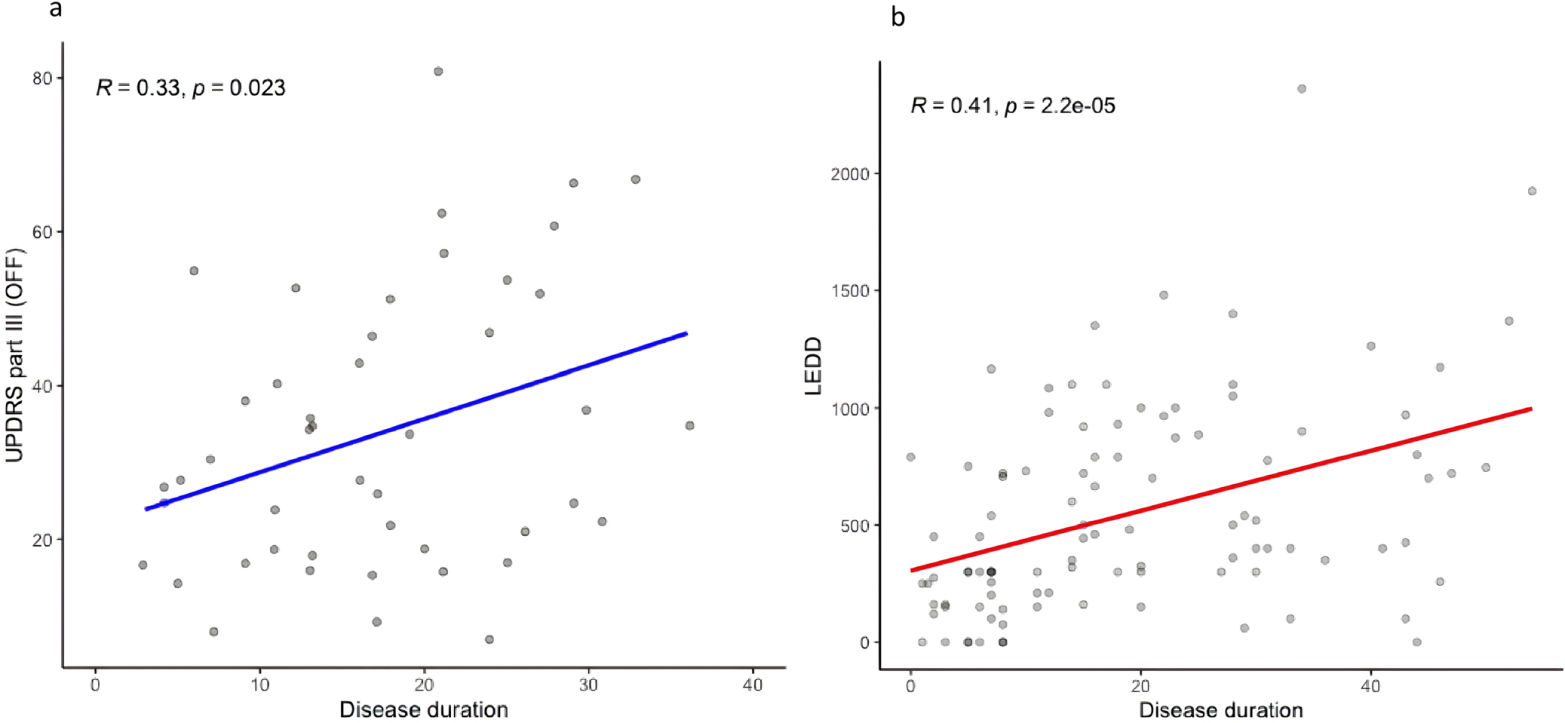
Linear regression models of motor progression. **a** UPDRS part III (OFF) scores at a given disease duration and (**b**) LEDD at a given disease duration.

The age at onset and sex influenced the UPDRS III (ON) score with age at onset increasing UPDRS III (ON) score by 2 points every 10 years, and men having 3.7 points more than women at all disease duration and ages of onset (Supplementary Fig. 6). The average UPDRS III (ON) score progression adjusted for age at onset and sex was estimated at 4.5 ± 0.6 points every 10 years based on the cross-sectional data (*n* = 343, *p* = 3.7e-11). Motor progression modelled by using longitudinal data of UPDRS III score at two different time points for the same individual, demonstrated that the ON scores increased by 3.7 ± 1.2 points every 10 years (*n* = 42, *p* = 0.005). The UPDRS III (OFF) score progression was estimated at 6.2 ± 2.7 points every 10 years in a subset of patients from whom this data was available (*n* = 51, *p* = 0.023) (Fig. 5a).

The probability of being in Hoehn and Yahr stage ≥3 was 17% (95% confidence interval (CI) 13–24%) at 10 years, 31% (95%CI 24–39%) at 20 years, and 58% (95%CI 45–73%) at 35 years (Supplementary table 5 and Supplementary Fig. 7).

### Motor Complications

**Fig. 6:**
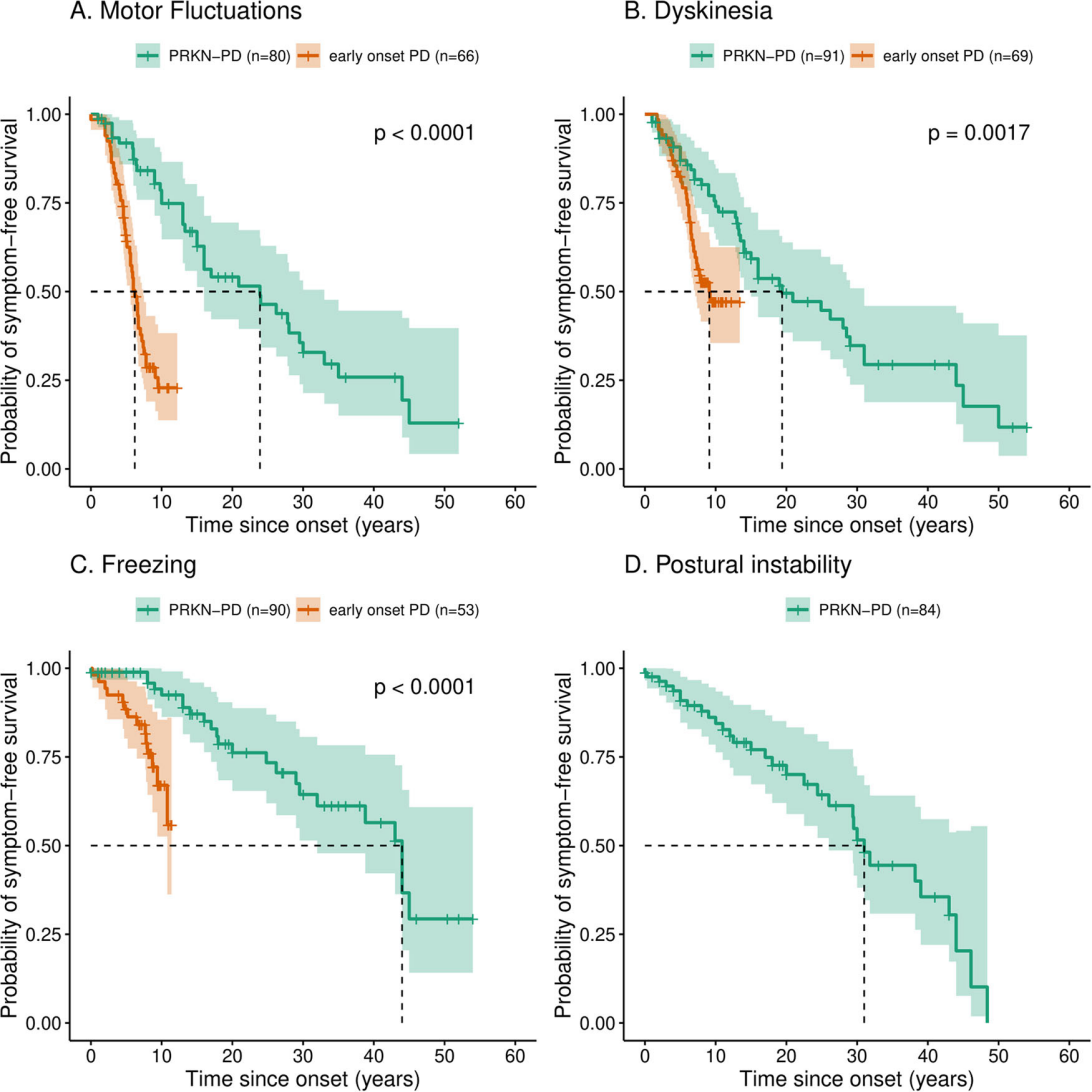
Kaplan-Meir survival curves comparing the time in years from first symptom to development of motor complications in *PRKN*-PD and early-onset PD. Time to develop (**A**) motor fluctuations, (**B**) dyskinesia and (**C**) freezing in *PRKN*-PD compared to early-onset PD. **D** Kaplan-Meir survival curve demonstrating the time in years from first symptom to development of postural instability in *PRKN*-PD. (The dashed lines delineate the time by which half of the patients had developed the complication).

The average age at onset of first symptom of PD in *PRKN*-PD patients with information about the prevalence of at least one feature of motor complications (*n* = 107) was 29.72 ± 11.34 years, in comparison to 43.53 ± 5.68 years in early-onset PD (*n* = 72) (Supplementary Fig. 8). The time in years since first symptom, at which half of the patients had developed motor complications was significantly later in those with *PRKN*-PD in comparison to those with early-onset PD. They were: 23.9 years (*n* = 80, 95%CI: 16 to 33) for motor fluctuations in *PRKN*-PD in comparison to 6.2 years (*n* = 72, 95%CI: 5.5 to 7.4) in early-onset PD; 19.4 years (*n* = 91, 95%CI: 15 to 31] for dyskinesia in *PRKN-*PD compared to 9.1 years (*n* = 74, 95%CI: 7.1 to N/A] in early-onset PD; 44 years (*n* = 90, 95%CI: 32 to N/A) for freezing in *PRKN*-PD and not reached in early-onset PD (*n* = 56) (Fig. 6, p < 0.05). The time to develop postural instability from first symptom was 31 years (*n* = 84, 95%CI: 26 to N/A) in *PRKN*-PD (Fig. 6).

### Levodopa daily dose and deep brain stimulation

In the 137 individuals for whom information was available about the presence or absence of deep brain stimulation (DBS), 23 had DBS in situ, with a mean disease duration of 23.7 ± 10.1 years at the time of DBS surgery (*n* = 20). Only four patients were treated with an apomorphine pen or pump (*n* = 107), while there were no patients with *PRKN*-PD (*n* = 105) treated with levodopa/carbidopa intestinal gel.

The evolution of LEDD scores with disease progression was modelled in individuals that never had DBS in situ (*n* = 114) to avoid the confounding effect of DBS. The average initial LEDD was 305 ± 68 mg, and this dose increased by 128 ± 28.7 mg every 10 years (*p* = 2.2e-5, Fig. 5b). Sex or age at onset of PD did not influence LEDD scores.

The evolution of LEDD scores based on disease duration, for those who had dystonia as their first symptom (*n* = 28), was not different to those who had tremor as their first symptom (*n* = 37, *p* = 0.09).

### Non-motor symptoms

The patients for whom phenotypic information was available, also described autonomic dysfunction, sleep disturbances, olfactory dysfunction and neuropsychiatric symptoms at the time of their last clinic visit (Table 2).

**Table 2:** Prevalence of non-motor symptoms in *PRKN*-PD (n = 152)^a^

Non-motor symptoms	Frequency	Present	Absent	Missing data
Autonomic dysfunction	62.6%	*n* = 72^b^	*n* = 43^c^	*n* = 37
Orthostatic hypotension	28.4%	*n* = 29	*n* = 73	*n* = 50
Constipation	37.7%	*n* = 40	*n* = 66	*n* = 46
Urinary urgency	47.7%	*n* = 53	*n* = 58	*n* = 41
Sleep disturbance	72.5%	*n* = 87^d^	*n* = 33^e^	*n* = 32
Somnolence	37.8%	*n* = 42	*n* = 69	*n* = 41
Insomnia	63.5%	*n* = 73	*n* = 42	*n* = 37
RBD	29.4%	*n* = 32	*n* = 77	*n* = 43
Sleep apnoea	11.5%	*n* = 9	*n* = 69	*n* = 74
Restless legs syndrome	13.9%	*n* = 15	*n* = 93	*n* = 44
Olfactory dysfunction	18.5%	*n* = 12	*n* = 53	*n* = 87
Impulse control disorders or punding	21.4%	*n* = 24	*n* = 88	*n* = 40
Psychological disturbance	64.5%	*n* = 82^f^	*n* = 45^g^	*n* = 25
Apathy	11.8%	*n* = 13	*n* = 97	*n* = 42
Depression	40.5%	*n* = 49	*n* = 72	*n* = 31
Anxiety	49.5%	*n* = 59	*n* = 60	*n* = 33
Hallucinations	18.5%	*n* = 23	*n* = 101	*n* = 28
Psychosis	9.3%	*n* = 10	*n* = 97	*n* = 45
Cognitive impairment	20.8%	*c* = 20^h^	*n* = 76^i^	*n* = 56
Mild cognitive decline^j^	24.4%	*n* = 19	*n* = 59	*n* = 74
Dementia	1%	*n* = 1	*n* = 95	*n* = 56

^a^Supplementary Fig. 5 depicts the flow chart demonstrating the number of patients included in assessing the prevalence of non-motor symptoms.

^b^Cases reporting the presence of any one of the following features of autonomic dysfunction: orthostatic hypotension, constipation or urinary urgency.

^c^Cases reporting the absence of all of the following features of autonomic dysfunction: orthostatic hypotension, constipation and urinary urgency.

^d^Cases reporting the presence of any one of the following features of sleep disturbance: somnolence, insomnia, RBD, sleep apnoea or restless legs syndrome.

^e^Cases reporting the absence of all of the following features of sleep disturbance: somnolence, insomnia, RBD, sleep apnoea and restless legs syndrome.

^f^Cases reporting the presence of any one of the following features of psychological disturbance: apathy, depression, anxiety, hallucinations or psychosis.

^g^Cases reporting the absence of all of the following features of psychological disturbance: apathy, depression, anxiety, hallucination and psychosis.

^h^Cases reporting the presence of mild cognitive decline or dementia.

^i^Cases reporting the absence of both mild cognitive decline and dementia.

^j^As defined by clinician’s opinion, or a MOCA < 26 or a MMSE < 25 in the 152 patients with detailed phenotypic information. Objective evidence of mild cognitive decline, with a MMSE < 25 (*n* = 253, Supplementary Fig. 1) was noted in 6% of cases.

Mild cognitive decline, as per the clinicians’ opinion or a Montreal Cognitive Assessment (MoCA) < 26 or a MMSE < 25 was reported in 24.4% patients (*n* = 78). However, there was only 6% (15*/* 253) of individuals with a MMSE score below 25 at examination. There was no correlation between the disease duration and the presence of cognitive impairment.

## Discussion

In this large multi-centre international cohort study, we reported variant-specific data on 647 patients with bi-allelic pathogenic *PRKN* variants. We categorised the mutational landscape in *PRKN*-PD, illustrated mutational hotspots and identified 20 variants that have not been previously reported. The large cohort size allowed us to clarify the type of variant and protein position location of variants that were associated with an earlier age at onset, a finding which has never been demonstrated before. This cohort was assessed at an average of 18 years from first motor symptom allowing for a deeper understanding of the progression of this illness. We described their motor and non-motor features, therapeutic choices and modelled their motor progression and time to development of motor complications.

Our findings suggest that the structure of the Parkin protein is important for its function with protein-truncating structural and frameshift variants and cumulative dysfunction of exons being associated with an earlier age at onset of the disease. The parkin protein is a RING-between-RING E3 ubiquitin ligase consisting of a ubiquitin-like domain and three RING domains (RING0, RING1 and RING2)^13,24–26^. The N-terminal ubiquitin-like domain binds to the RING1 domain. The ubiquitin-like domain and the RING1 domain were mutational hotspots, with variants located in the N-terminus being associated with the earliest age at onset, compared to variants located in the middle of the protein or the catalytic domain in the C-terminus of the protein. Although there was a predominance of frameshift variants in the N-terminus which could have contributed to the earlier age at onset, the high prevalence of variants in these 2 domains which interact with each other, does imply that dysfunction of Parkin’s role in substrate recognition and ubiquitin conjugation contributes to the resulting pathophysiology of PD^25^. However, we could not find an association between the age at onset and protein domain location of the variants, higher CADD scores suggestive of more deleterious single nucleotide variants or location of missense variants in a particular protein domain, suggesting that there is not just one functional domain that is most important in the protein. This is further demonstrated by the fact that the mutational landscape of *PRKN* is characterised by loss of function pathogenic variants that can affect every single exon and protein domain, highlighting the importance of all domains in its function. We have not considered the 3-dimensional crystal structure of Parkin and it is possible that damage to this structure characterised by greater dysfunctional exons, irrespective of the location of these variants, is more important for its function than individual protein domains^27^.

Prior genotype-phenotype studies in *PRKN*-PD were based on small cohorts with ambiguous results^20,21^. The first one conducted in 36 familial and 12 sporadic cases did not find genotype/phenotype correlation^20^. The second one in a small cohort of 25 patients found that point mutations were associated with an earlier age at onset in comparison to two exon deletions^21^. A previous case report has indicated that *PRKN*-PD with onset below the age of 10 is associated with N-terminal Parkin deletion^28^. A recent study assessing *PRKN* variants suggested that individuals with a single copy number variant in *PRKN* could have a greater risk for developing IPD and an earlier mean age at onset, in comparison to those that carried a single nucleotide variant^29^. However, it must be noted that *PRKN* is a very large gene and that a cryptic second variant could have been missed in this study involving heterozygous *PRKN* variants, since the entire gene is often not assessed completeley^29^. Therefore, this result suggestive of the more benign effect of single nucleotide variants, could in fact have been related to the effect in bi-allelic *PRKN*-PD and therefore consistent with our results.

We were unable to find variant-specific features associated with motor progression, but it must be noted that our modelling of motor progression was predominantly based on cross-sectional data which will bias the results. We also did not have the statistical power to determine genotype-specific features associated with the development of motor and non-motor complications. However, the finding that missense variants are associated with a later age at onset, and that individuals with a later age at onset have higher UPDRS III (ON) scores could suggest that individuals with missense variants have a phenotype more consistent with IPD, with a later age at onset and higher UPDRS III (ON) scores. Furthermore, the finding that women with two missense variants have a later age at onset is reminiscent of results in IPD. In IPD, men have twice as high a risk of developing the disease in comparison to women, while later age at onset of menopause is associated with an older age at onset of PD and better UPDRS III (ON) scores in women^30,31^. Separate to this hypothesis for these findings in women with missense variants, sex on its own is an independent influencer of motor scores in *PRKN*-PD, with women having lower UPDRS III (ON) scores compared to men.

This large cohort allowed us to model motor progression in *PRKN*-PD: UPDRS III (ON) scores increase by 0.45 points per year and UPDRS III (OFF) scores increase by 0.62 points every year. This rate of progression is exceptionally slow in comparison to the estimated increase in UPDRS III (ON) scores of 1.8 points per year in IPD^32^. Our results are consistent with a previous small longitudinal cohort study showing a rate of progression of UPDRS III (OFF) scores of 0.203 points per year in early onset *PRKN*-PD patients (age at onset less than 50 years), compared to 1.056 points per year in early onset genetically undiagnosed PD patients^12^. Our cohort was larger, however the motor progression in the (OFF) state was not assessed longitudinally but modelled from cross-sectional data and we did not have a control IPD population for direct comparison of progression rate. Age at onset in our cohort was not limited to less than 50 years and patients had longer disease duration, which might have contributed to the slightly higher rate of progression detected in our study with respect to this previous study. Our data, however, confirms that motor progression in *PRKN*-PD is significantly slower than previously described for IPD populations, consistent with the previously reported lower mortality rate^33^.

An earlier age at onset of PD was previously associated with the development of levodopa related motor complications such as dyskinesia^34^. *PRKN*-PD is characterised by severe dopaminergic neuronal loss in the substantia nigra pars compacta and therefore, it would be expected that these patients might have early development of levodopa related and unrelated motor complications^35^. In a large cohort of idiopathic PD patients, at 10 years after onset, 100% had motor fluctuations and 55.7% had levodopa-induced dyskinesias^36^. In our cohort of *PRKN*-PD patients, 49% (39/80) had developed motor fluctuations, with half developing it by 24 years of disease duration, which is in comparison to as early as 6 years in early-onset PD. 49% (45/91) of *PRKN*-PD patients had developed dyskinesias, with half developing it by 19 years, compared to half developing it by 9 years in early-onset PD. The *PRKN*-PD patients had an earlier age at onset of first symptom of PD, of an average of 30 years in comparison to 44 years in those with early-onset PD. We were unable to compare the time to develop motor complications with a cohort matched in age at onset of first symptom, however in this cohort that is as comparable as possible, the significant delay in development of motor complications in *PRKN*-PD is consistent with the slow motor progression of *PRKN*-PD and suggestive of protective compensatory mechanisms. A potential hypothesis is that *PRKN*-PD begins in childhood/ early adolescence at a time when the dopaminergic system has not completed development, and therefore it is more amenable to developing compensatory mechanisms in comparison to genetically undiagnosed early onset PD, which begins following maturation of the dopaminergic system^37,38^. However, an age-matched cohort of patients with genetically undiagnosed PD is necessary to confirm if the age at onset is the predominant compensatory factor or if there are other factors that are specific to Parkin.

One potential protective factor to the development of levodopa-induced dyskinesia could be the minimal requirement for an increase in dopaminergic medications with progression (118 ± 30 mg every 10 years in our cohort)^32^. Furthermore, it appears that those with tremor as their first symptom of *PRKN*-PD do not require higher doses of levodopa^39^. The good levodopa response in *PRKN*-PD despite the marked degree of nigrostriatal denervation is surprising and warrants further investigation to understand the neuroanatomical mechanisms involved. There were no patients in this cohort who required treatment with levodopa/carbidopa intestinal gel, perhaps due to this good response to low doses of levodopa along with the tendency to prefer DBS instead of levodopa/carbidopa intestinal gel in these patients, given their younger age.

*PRKN*-PD is thought to be a motor-predominant disease and it is well known that cognition is preserved in these patients. However, the impact on other non-motor features has not been well studied^7,11^. There was a 21.4% prevalence of impulse control disorders (ICDs) or punding in this cohort. A prior study comparing ICDs in *PRKN*-PD to early onset genetically undiagnosed PD demonstrated a similar prevalence between the two groups but a higher frequency and severity of specific impulse control disorders including compulsive shopping, binge eating and punding in *PRKN*-PD^40^. Therefore, appropriate counselling and caution is necessary with the use of dopamine agonists in this cohort.

Our results also demonstrate the high prevalence of non-motor symptoms including autonomic dysfunction (more than 25%), rapid eye movement (REM) sleep behaviour disorder (RBD) (29%) and hyposmia (18.5%), which is higher than prior reports in the literature. However, in our study we included cases with clinical suspicion of these features without formal confirmation through tests such as polysomnography, which could have contributed to the high prevalence. Autonomic dysfunction, olfactory dysfunction and sleep disorders were only noted in 2–4% in a previous report of *PRKN*-PD patients, but there was up to 95% missing data in that cohort^7^. Another cohort of *PRKN*-PD patients noted comparable rates of autonomic dysfunction to our findings with 20.8% suffering from orthostatic hypotension, 30.2% with constipation and 17.5% with urinary incontinence^41^. However only 2.7% had olfactory dysfunction and nobody had RBD in this cohort^41^. Hyposmia and RBD are considered to be prodromal markers for IPD and thereby alpha-synuclein pathology^10,13,42–44^. Our finding that 29% of *PRKN*-PD patients report symptoms of RBD is interesting since *PRKN*-PD is not typically considered to be a synucleinopathy^10^. However, it is possible that RBD in *PRKN*-PD is secondary to neurodegeneration of the locus coeruleus^45^, with lesions in this region associated with REM sleep without atonia^46^. In a prior study involving a large cohort of patients with idiopathic RBD, there were no cases with pathogenic bi-allelic *PRKN* variants identified, however this could perhaps be because RBD is not a prodromal symptom in *PRKN*-PD, but rather a marker of progression of the disease^47^. On the other hand, the prevalence of hyposmia in 18.5% of our cohort warrants investigation as to whether this is related to underlying alpha-synuclein pathology^48^. These findings highlight the need for longitudinal assessment of non-motor symptoms in *PRKN*-PD to understand if these are prodromal symptoms or a marker of progression of the illness, thereby allowing us to gain insight into the neuroanatomical regions involved by this disease.

The limitations of this study are inherent to its cross-sectional nature and to the data collection occurring in a real-life setting. Missing data were present with different information available at different centres and from different databases. Full phenotype information was only present for less than 21% of the cohort. We lacked information on the longitudinal evolution of non-motor symptoms or the contribution of treatments to these symptoms e.g. the influence of dopaminergic medications on orthostatic hypotension or anticholinergics on constipation. The absence of information on which individuals in our cohort had confirmation of non-motor features through objective assessments such as polysomnography and validated tests to assess olfactory performance, limits the generalisability of this data and necessitates prospective studies dedicated to investigating non-motor symptoms in *PRKN*-PD. We focused the principal analysis on age at onset, which was available for most cases. However, further prospective longitudinal studies are needed to investigate genotype-phenotype correlation with disease progression. Furthermore, we classified the variants into the categories missense, frameshift or structural, however, some structural variants can affect the reading frame and result in a truncated protein similar to a frameshift variant and therefore this classification could result in potential bias. The bi-allelic nature of the pathogenic variants was not verified for 47% of cases leading to potential inclusion bias. The sample size was small for modelling the evolution of treatment (LEDD and advanced therapies). Our cohort of early-onset PD patients all had testing for pathogenic variants in *LRRK2* and *GBA*, but they did not all undergo thorough investigation for pathogenic variants in other genes, and therefore these results should be interpreted with caution. Finally, although our study collected data from 46 different countries, 76.5% of patients were Caucasian and therefore, our study lacks information about variants found in other ethnicities, in particular the Asian and African population.

In conclusion, our work on the mutation-specific and clinical features of *PRKN*-PD contributes important insights into this disease. On a genetic level, we demonstrated that age at onset relates to the type and location of variants, probably through the degree of structural integrity of the protein and the level of residual enzymatic activity. The wide distribution in age at onset in individuals that possess the same variant is however suggestive of other genomic, epigenetic or environmental modifiers of phenotype. Given that exon 3 deletions are the most common variant in this and prior cohorts, modulators of splicing may be considered as a potential therapy for *PRKN*-PD^7,49^. Furthermore, since the ubiquitin-like domain is a mutational hotspot, potential targeted therapies should be developed to counteract for the loss of function of this domain. On a clinical level, we confirm the slow motor progression, minimal increase in levodopa dose and late development of motor complications, highlighting the need for other biomarkers for end points in future clinical trials. Further studies are needed to confirm our results and better apprehend the progression of motor and non-motor symptoms in a longitudinal, prospective manner.

## Methods

## Population

Patients with bi-allelic pathogenic variants in *PRKN* and clinically diagnosed PD were included from three main sources. Firstly, data from the MJFF Global Parkinson’s Genetics Program was filtered to identify individuals with pathogenic bi-allelic (compound heterozygous or homozygous) variants in *PRKN*^50^. Patients were confirmed to have pathogenic variants in *PRKN* using either candidate gene sequencing involving a PD panel, Multiplex Ligation dependent probe amplification or a single nucleotide polymorphism (SNP) array. Secondly, data from the NGC/ NS-Park study which is a long-standing French Cohort Study on patients with PD, with collaborations from Mediterranean countries, including Turkey, Algeria and Tunisia was filtered to identify patients with 2 pathogenic variants in *PRKN*^22^. Lastly, a multi-centre collaborative study, GPiP was commenced with data obtained from 12 centres (Supplementary Table 6) on PD patients with bi-allelic pathogenic variants in *PRKN*.

Patients with either one pathogenic variant in an autosomal dominant gene known to be associated with PD, or two pathogenic variants in another autosomal recessive gene, were excluded from the analysis. Segregation studies were not always possible for all patients included in NGC and GPiP, therefore the bi-allelic nature of these variants was assumed by the location of the variants in the gene, i.e., if two single nucleotide variants were located on different exons, it was presumed that they were bi-allelic in nature. On the other hand, copy number variants that were located beside each other (e.g., an exon 2 and an exon 3 deletion) were considered to be mono-allelic.

The prevalence of variants was assessed only in index cases (*n* = 582), while all other association studies were undertaken from the entire cohort of 647 patients with bi-allelic PRKN PD (Supplementary Fig. 2).

PD patients being followed up in a multicentre longitudinal cohort study, Drug Interaction with Genes in Parkinson’s disease, were filtered to identify those who had first symptom before the age of 50^51^. Six of these patients were shown to have either pathogenic variants in *GBA* or the G2019S variant in *LRRK2* and were excluded. 72 patients were included in the comparison cohort with early-onset PD.

### Ethics

All patients included had given their informed consent, and DNA collection was undertaken as part of a clinical study that had received approval from ethics committees in each centre. The NGC/ NS-Park study was approved by the French National Institute of Health and Medical Research (INSERM).

### Genetic variants

All cases included had information about the two *PRKN* variants (reference sequence NM_004562.3). Variants identified through the different sources were checked for concordance with the variants identified in *PRKN* in MDSGene (https://www.mdsgene.org/) and in the previous publication by Lesage et al. ^22^. The pathogenicity of variants that were not present in these two sources were analysed using Alamut and Varsome, according to the American College of Medical Genetics and Genomics criteria^52^. CADD scores were determined for all single nucleotide variants and insertions/deletions. SpliceAI scores were used to predict if an intronic variant has a splice effect^53^. The bi-allelic nature of the pathogenic variants (whether they were in *cis* or *trans*) was not systematically assessed for all cases but was verified for 53% of patients. Individuals carrying pathogenic variants in any other gene associated with monogenic PD (e.g., heterozygous *LRRK2* pathogenic variants or homozygous *PINK1* variants) were excluded.

The variants in *PRKN* were classified based on the zygosity, type of variant (structural, frameshift, missense, nonsense, or splice site variant), the number of exons altered or absent secondary to the variant (structural variants which preserved the reading frame e.g. deletion of exon 5 was calculated to affect just the one exon, while truncating structural and frameshift variants, nonsense variants and splice site variants were calculated to affect all the exons lost secondary to the variant), the exonic location, and the protein domain involved (ubiquitin-like, RING0, RING1, in-between- RING (IBR), RING2).

### Clinical characteristics

Individual level data about the sex of the participant, self-reported ethnicity (European, North African, African/Black, East-Asian, South-Asian, other), country of origin and whether other family members were diagnosed with PD was available.

Phenotypic characteristics of this population, including age at onset, motor severity (UPDRS part III (UPDRS III) score and Hoehn and Yahr scale in the on- (ON) and off- (OFF) state), cognition (MMSE score), and LEDD were assessed cross-sectionally and longitudinally (Supplementary Fig. 2). Movement Disorder Society-Sponsored Revision of the Unified Parkinson’s Disease Rating Scale (MDS-UPDRS) part III scores were converted into UPDRS III scores as previously reported for individuals that had Hoehn and Yahr stages available^54^ and for individuals without Hoehn and Yahr stages available^55^. Similarly, MoCA scores were converted into MMSE equivalent scores as previously suggested given the greater availability of the latter in our dataset^56^.

Detailed phenotypic information was available for patients from 11 GPiP centres (*n* = 129) and 23 patients from NGC (Supplementary Fig. 5). This information consisted of initial motor symptoms and the time interval from first motor symptom to diagnosis as PD. The presence or absence of motor complications (as defined by the clinician’s opinion or the MDS-UPDRS part II, III and IV) including motor fluctuations, dyskinesia, freezing, postural instability and the time interval to develop these complications from the first motor symptom was available for a subset (Supplementary Fig. 5). The time to develop these motor complications, other than postural instability, were also available for the comparison cohort of patients with early-onset PD.

The presence or absence of non-motor symptoms, as defined by the clinician’s opinion or suggested by clinical history, or self-reported by the patient through the MDS-UPDRS part I, or assessed through validated tests such as the University of Pennsylvania Smell Identification Test (UPSIT), Brief Smell Identification test (BSIT) or Sniffin’ sticks test (for olfactory dysfunction), MoCA or MMSE (for cognition) or polysomnography (for RBD) were available for these 152 patients (Supplementary Fig. 5). The non-motor symptoms that were assessed included: olfactory dysfunction, orthostatic hypotension, constipation, urinary urgency, somnolence, insomnia, RBD, restless legs syndrome, sleep apnoea, apathy, depression, anxiety, hallucinations, psychosis, ICDs or punding, mild cognitive impairment and dementia (Supplementary Fig. 5).

Information about treatment at time of examination, including the presence or absence of advanced PD therapies such as DBS, apomorphine pump and levodopa/carbidopa intestinal gel were also accessible.

### Statistical analysis

Continuous data were reported as mean ± standard deviation or median with interquartile ranges for normal and non-normal data, while categorical data were expressed as numbers and percentages. Patients’ characteristics within and between cohorts were compared by two-sample t-test, one-way analysis of variance (ANOVA) and Chi-square test as appropriate. Post hoc comparisons were performed using pairwise Chi-square tests with Benjamini Hochberg correction for categorical variables and Tukey HSD tests for numerical variables.

Multiple linear regression and logistic regression analyses were used to compare phenotypic features with variant characteristics. The predefined primary clinical criterion was the age at onset, and the predefined classification features of the genetic variants were the type of variant, total number of exons affected by the two variants, the protein location and the protein domains involved by the variant. Main secondary criteria were the Hoehn and Yahr (ON), and the UPDRS part III (ON), which were available for most patients. Models were fitted to study the associations: between the age at onset and the type of variant (first model), the total number of exons affected (second model), the protein location of variants (third model), the protein domains involved by the variants (fourth model) and between Hoehn and Yahr (ON) and UPDRS part III (ON) and the type of variant (fifth and sixth model), the total number of exons affected by the two variants (seventh and eight model) and the protein location of variants (ninth and tenth model). Models were adjusted for sex (all models), and disease duration (models 5–10) and their interaction with features of the genetic variants were tested in supplementary analyses. Significance of the main and interaction effects was assessed based on type II Wald Chi-square tests using the ‘Anova’ function in the ‘car’ R package, followed by post hoc pairwise comparisons using the ‘emmeans’ R package with Tukey’s adjustment for multiple testing.

For modelling disease progression, a linear regression analysis was performed using UPDRS III or LEDD scores at each time point (disease duration) for each patient with age at onset and sex as covariates. Kaplan-Meier symptom-free survival curves were generated for the time since the first symptom to the development of motor complications using the ‘survival’ version 3.5–7 and ‘survminer’ version 0.4.9R packages. In the patients with early-onset PD who had information available on their time to develop motor complications, their symptom-free survival curve was compared to the *PRKN*-PD survival curve using the log-rank test.

Considering the exploratory nature of this work, the level of statistical significance was set at *p* value < 0.05 for all tests. No imputation was performed for missing data. Analyses were run only in patients with all data available for each analysis. The flow charts depict the number of patients included for each section of the analysis for the main models (Supplementary Figs. 2 and 5). The number of patients used for the analysis is also provided. The distribution of the age at onset and the sex ratio was verified to be similar between the subgroup of patients used for the separate analysis and the entire cohort.

### Reporting summary

Further information on research design is available in the Nature Research Reporting Summary linked to this article.

### Supplementary information

Supplementary Material

Reporting Summary

## Data Availability

The data that support the findings of this study are available on request from the corresponding author. The data are not publicly available, as it contain information that could compromise the privacy of study participants.

## Code availability

All statistical analyses were conducted using R version 4.1.2 (R Development Core Team, 2021) and the R codes used are available on request from the corresponding author.
