# Supplementary material for: Genotype–phenotype correlation in PRKN-associated Parkinson’s disease
Source: NPJ Parkinsons Dis. 2024 Mar 29;10:72. doi: 10.1038/s41531-024-00677-3 (PMC10980707; doi:10.1038/s41531-024-00677-3)
Supplement: Supplementary file 1 — Supplementary Material [file 41531_2024_677_MOESM1_ESM.pdf]

Supplementary figure 1: Histogram of age at onset of *PRKN*-PD in the cohort.

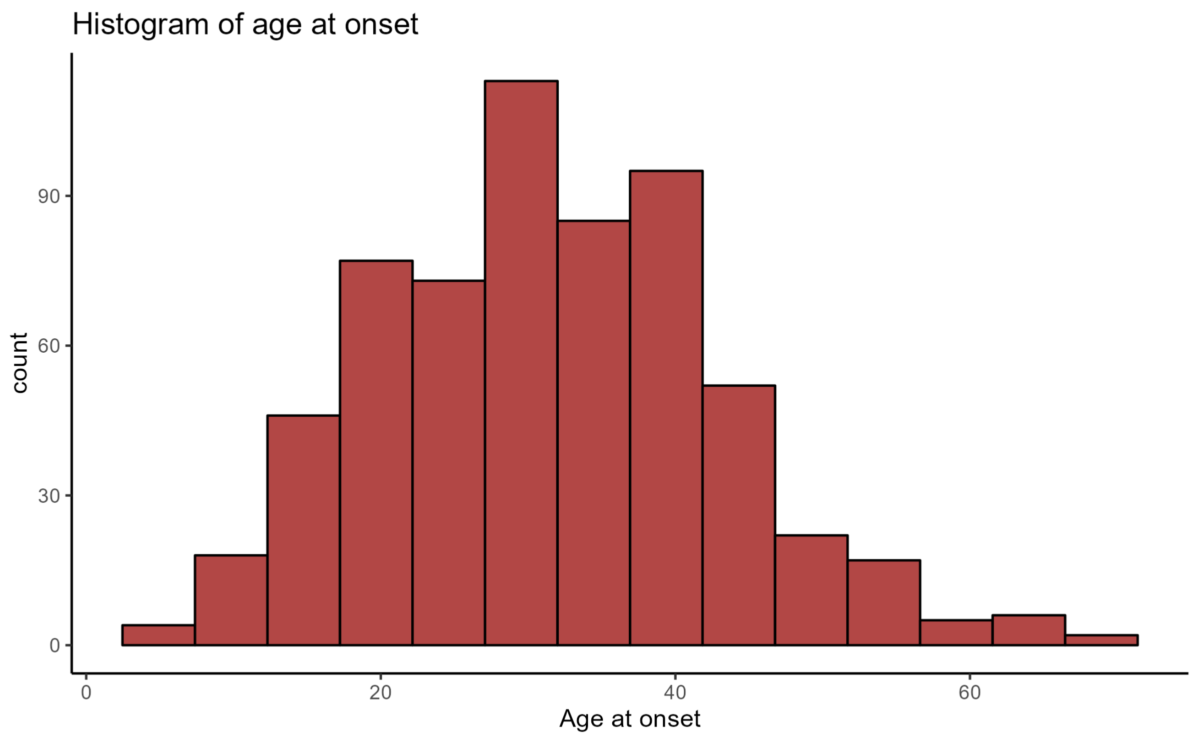

Supplementary table 1: Demographic and clinical characteristics of the NGC, MJFF and GPiP centres.

	NGC (a)	MJFF (b)	GPiP centres (c)	P value‡
**Number of patients**	227 (35.1%)	253 (39.1%)	167 (25.8%)	
**Female : Male**	110 : 104 *(n=214)*	124 : 129 *(n=253)*	74 : 91 *(n=165)*	0.4
**Mean age at onset (± SD)**	30.7 ± 10.7 *(n=205)*	32.4 ± 11.2 *(n=245)*	30.9 ± 12.3 *(n=165)*	0.23
**Mean disease duration (± SD)**	16.0 ± 11.1 c *(n=144)*	18.4 ± 11.7 *(n=219)*	20.2 ± 14.6 a *(n=140)*	0.016*
**Mean UPDRS part III (ON) (± SD)**	17.4 ± 13.6 b *(n=124)*	23.1 ± 17.2 a *(n=138)*	19.1 ± 12.9 *(n=92)*	0.008*
**Mean UPDRS part III (OFF) (± SD)**	33.4 ± 17.2 *(n=58)*	N/A	36.0 ± 2.8 *(n=2)*	0.8
**Mean Hoehn and Yahr (ON) (± SD)**	1.9 ± 1.0 b, c *(n=127)*	2.2 ± 0.9 a *(n=149)*	2.2 ± 0.9 a *(n=81)*	0.003*
**Mean Hoehn and Yahr (OFF) (± SD)**	2.9 ± 1.3 c *(n=41)*	N/A	2.3 ± 1.1 a *(n=28)*	0.03*
**Mean MMSE (± SD)**	28.6 ± 3.3 *(n=106)*	27.4 ± 4.9 c *(n=67)*	28.9 ± 1.8 b *(n=80)*	0.02*
**Mean LEDD (mg) (± SD)**	430.2 ± 317.8 c *(n=141)*	N/A	587.7 ± 574.2 a *(n=111)*	0.006*
**Presence of DBS**	10 *(n=27)*	N/A	13 *(n=110)*	0.004*
**Mean disease duration at time of DBS in years (± SD)**	26.0 ± 8.7 *(n= 9)*	N/A	21.7 ± 11.1 *(n= 11)*	0.4

Notes. Following letters indicate which groups significantly differ: a group differs from NGC cohort; b group differs from MJFF cohort; c group differs from GPiP centres.
‡ Chi-square test was used to compare the groups for categorical variables and One way ANOVA for numerical variables. Post hoc comparisons were performed using pairwise Chisquared tests with Benjamini Hochberg correction for categorical variables and Tukey HSD tests for numerical variables.

Supplementary table 2: Pathogenic *PRKN* variants present in index cases in the cohort.

The number refers to the number of times the variant was encountered in index cases. (If the variant was present on both alleles, e.g., a homozygous exon 3 deletion, it has been accounted for twice in the number count).
CADD scores have been included for single nucleotide variants.
CADD scores are not applicable for structural and frameshift variants.
SpliceAI maximum delta scores have been included for intronic variants.

	**Variant**	**Number**	**% of index cases**	**CADD scores**	**SpliceAI score**
1	deletion of exon 3	145	12.3		
2	R275W	117	10.0	26.1	
3	deletion of exon 3,4	80	6.8		
4	N52Mfs*29	79	6.7		
5	deletion of exon 4	65	5.5		
6	Q34Rfs*5	50	4.3		
7	deletion of exon 2	43	3.7		
8	deletion of exon 5	41	3.5		
9	duplication of exon 3	29	2.5		
10	G430D	26	2.2	25.8	
11	P113Tfs*51	25	2.1		
12	deletion of exon 5,6	22	1.9		
13	deletion of exon 7	20	1.7		
14	deletion of exon 3,4,5,6	15	1.3		
15	E395*	14	1.2	36	
16	C253Y	13	1.1	26.3	
17	R42P	13	1.1	24.9	
18	deletion of exon 2,3,4	13	1.1		
19	deletion of exon 2,3	11	0.9		
20	deletion of exon 8,9	11	0.9		

21	deletion of exon 6	11	0.9		
22	V324Afs*111	10	0.9		
23	K211N	9	0.8	24.9	
24	P437L	9	0.8	28.1	
25	deletion of exon 1	9	0.8		
26	R402C	8	0.7	25.1	
27	T240M	8	0.7	23.4	
28	W74Cfs*8	8	0.7		
29	deletion of exon 3,4,5	8	0.7		
30	deletion of exon 4,5	8	0.7		
31	deletion of promotor and exon 1	7	0.6		
32	C212Y	6	0.5	26.9	
33	C238W	6	0.5	23.6	
34	C289G	6	0.5	27	
35	C441R	6	0.5	29.4	
36	V56E	6	0.5	27.4	
37	c.1286-3C>G	6	0.5	23	Acceptor loss 0.87
38	deletion of exon 6,7	6	0.5		
39	deletion of exon 8	6	0.5		
40	duplication of exon 4,5,6	6	0.5		
41	duplication of exon 7	6	0.5		
42	G284R	5	0.4	25.3	
43	R334C	5	0.4	18.9	
44	W445*	5	0.4	44	
45	deletion of exon 5,6,7	5	0.4		
46	duplication of exon 11	5	0.4		
47	duplication of exon 2,3,4	5	0.4		
48	triplication of exon 2	5	0.4		
49	E79*	4	0.3	33	
50	F362Lfs*73	4	0.3		
51	K27del	4	0.3		
52	M1?	4	0.3		
53	Q311*	4	0.3	57	
54	R33*	4	0.3	45	
55	C268R	3	0.3	28.2	
56	H303Y	3	0.3	26.7	
57	R234Q	3	0.3	20.9	
58	R256C	3	0.3	32	
59	T415N	3	0.3	25.2	
60	deletion of exon 11	3	0.3		

61	deletion of exon 4,5,6,7	3	0.3		
62	duplication of exon 2	3	0.3		
63	duplication of exon 4	3	0.3		
64	duplication of exon 5	3	0.3		
65	duplication of exon 6	3	0.3		
66	duplication of exon 7,8	3	0.3		
67	S198Pfs*27	2	0.2		
68	deletion of exon 4,5,6	2	0.2		
69	*466Yext*24	2	0.2		
70	duplication of exon 9	2	0.2		
71	C201Mfs*5	2	0.2		
72	C212Wfs*13	2	0.2		
73	D243N	2	0.2	23.4	
74	H257R	2	0.2	25.8	
75	P133Qfs*44	2	0.2		
76	P159L	2	0.2	25.6	
77	Q178*	2	0.2	47	
78	R275Q	2	0.2	31	
79	c.1083+1delG	2	0.2	32	Donor loss 1.00 Donor gain 1.00
80	c.535-2A>C	2	0.2	32	Acceptor loss 0.71
81	c.7+1G>A	2	0.2	33	Donor loss 0.96
82	deletion of exon 1,2	2	0.2		
83	deletion of exon 1,2,3	2	0.2		
84	deletion of exon 1,2,3,4,5,6	2	0.2		
85	deletion of exon 10,11,12	2	0.2		
86	deletion of exon 12	2	0.2		
87	deletion of exon 2,3,4,5	2	0.2		
88	deletion of exon 4,5,6,7,8,9	2	0.2		
89	deletion of exon 7,8,9	2	0.2		
90	duplication of exon 3,4,5	2	0.2		
91	duplication of exon 4,5,6,7	2	0.2		
92	duplication of exon 6,7	2	0.2		
93	A225Ffs*8	1	0.1		
94	A46T	1	0.1	25.4	

95	C150*	1	0.1	41	
96	C166Hfs*18	1	0.1		
97	C166Y	1	0.1	24.9	
98	C212G	1	0.1	28.3	
99	C352R	1	0.1	25.3	
100	D130AH	1	0.1		
101	D53E	1	0.1	22.9	
102	D87Tfs*16	1	0.1		
103	E310D	1	0.1	18.74	
104	H433P	1	0.1	28.2	
105	I29Cfs*27	1	0.1		
106	N428Kfs*141	1	0.1		
107	N428S	1	0.1	24.5	
108	P132Tfs*9	1	0.1		
109	Q100*	1	0.1	22	
110	Q100H	1	0.1	11.36	
111	Q276Rfs*22	1	0.1		
112	Q376Sfs*59	1	0.1		
113	R33Q	1	0.1	18.7	
114	W453*	1	0.1	44	
115	c. 735-2A>G	1	0.1	33	Acceptor loss 1.0
116	c.1084-1G>A	1	0.1	25.7	Acceptor loss 1.0
117	c.1084-1G>C	1	0.1	25.1	Acceptor loss 0.99
118	c.534+3A>G	1	0.1	19	Acceptor loss 0.48
119	c.7+1G>T	1	0.1	33	Donor loss 0.96
120	c.7+5G>T	1	0.1	23	Donor gain 0.53
121	deletion of exon 10	1	0.1		
122	deletion of exon 3,4,5,6,7	1	0.1		
123	deletion of exon 5,6,7,8,9	1	0.1		
124	deletion of exon 7,8	1	0.1		
125	duplication of exon 1,2	1	0.1		
126	duplication of exon 12	1	0.1		
127	duplication of exon 2,3	1	0.1		
128	duplication of exon 2,3,4,5	1	0.1		
129	duplication of exon 2,3,6	1	0.1		

130	duplication of exon 3,4,5,6,7,8,9	1	0.1		
131	duplication of exon 5,6,7	1	0.1		
132	duplication of exon 7,8,9,10,11,12	1	0.1		
133	triplication of exon 3	1	0.1		

Supplementary table 3: Variants that have not previously been reported that were identified in this cohort.

Variant	Source	2nd variant with it	ACMG classification	ClinVar	Age at onset	Reason for inclusion
Q100H	GPiP	deletion of exon 3,4	Likely benign	Uncertain significance	50	Not present in gnomAD and confirmation from center that variant is responsible for phenotype
P159L	GPiP	homozygous	VUS		56	Rare variant with MAF of 0.00003 and confirmation from center that variant is responsible

						for phenotype
H303Y	MJFF	2 cases with R275W and another case with deletion of exon 5,6	VUS	Not in clinvar	53, 43 and 38	Rare variant (MAF 0.000013) with known pathogenic second variant
R275Q	MJFF	With R275W	Likely pathogenic	Uncertain significance	46	Likely pathogenic variant
P133Qfs*44	MJFF	Homozygous	Likely pathogenic	Not in ClinVar	33	Likely pathogenic variant
c.534+3A>G	MJFF	D53E	VUS	Not in ClinVar	38	Rare splice region variant (MAF 0.0000197)
C150*	MJFF	Deletion of exon 2,3	Likely pathogenic	Not in ClinVar	40	Likely pathogenic variant
C166Hfs*18	GPiP	G284R		Not in ClinVar	28	Variant associated with loss of function
C166Y	GPiP	Deletion of exon 1,2,3	Likely pathogenic	Not in ClinVar	17	Likely pathogenic variant
C352R	GPiP	R275W	VUS	Not in ClinVar	34	Variant not present in gnomAD
c.388_389insCAC (p.Asp130delinsAlaHis)	GPiP	Deletion of exon 3		Not in ClinVar	48	Variant not present in gnomAD

D53E	MJFF	c.534+3A>G	VUS	Not in ClinVar	38	Variant not present in gnomAD
c.259del (p.Asp87ThrfsTer16)	GPiP	Deletion of exon 4	Likely pathogenic	Not in ClinVar	47	Variant not present in gnomAD
H433P	NGC	deletion of exon 3,4,5,6	VUS	Not in ClinVar	41	Variant not present in gnomAD
I29Cfs*27	NGC	c.7+1G>A		Not in ClinVar	24	Variant associated with loss of function
N428S	MJFF	deletion of exon 3,4	VUS	Not in ClinVar	44	Variant not present in gnomAD
P132Tfs*9	GPiP	P437L		Not in ClinVar	66	Variant associated with loss of function
Q100*	GPiP	R275W	Likely pathogenic	Not in ClinVar	19	Likely pathogenic variant
Q376Sfs*59	NGC	deletion of exon 3,4		Not in ClinVar	39	Variant associated with loss of function
C212G	GPiP	Homozygous deletion of exon 5,6	Likely pathogenic	Not in ClinVar	38	Likely pathogenic variant

(VUS = variant of uncertain significance)

Supplementary table 4: Molecular features of *PRKN* variants identified in index cases.

Feature	N	Proportion
**Zygosity**		
Compound Heterozygous	319	54.8%
Homozygous	263	45.2%
**Type of variant (Variant 1/ Variant 2/ Variant 3)**		
Structural/ Structural	228	39.2%
Missense / Structural	103	17.7%
Structural/ Frameshift	60	10.3%
Missense/ Missense	56	9.6%
Frameshift/ Frameshift	49	8.4%
Missense/ Frameshift	36	6.2%
Nonsense/ Nonsense	13	2.2%
Nonsense/Structural	6	1.1%
Missense/ Splice site	5	0.9 %
Splice site/ Splice site	4	0.7%
Missense/ Missense/ Missense	3	0.5%
Nonsense/ Missense	3	0.5%
Structural/ Structural/ Structural	3	0.5%
Indels/ Indels	2	0.3%
Splice site/ Structural	2	0.3%
Missense / Missense / Structural	2	0.3%
Frameshift/ Splice site	1	0.2%
Missense / Frameshift / Structural	1	0.2%
Missense/ Missense/ Splice site	1	0.2%
Structural / Structural / Missense	1	0.2%
Structural/ Indels	1	0.2%
Frameshift/ Nonsense	1	0.2%
**Most frequent exonic location of variants***		
Exon 3/ Exon 3	61	10.5%
Exon 2/ Exon 2	56	9.6%
Exon 2/ Exon 3	28	4.8%
Exon 7/ Exon 7	26	4.5 %
Exon 3,4/ Exon 3,4	21	3.6%

Exon 4/ Exon 4	18	3.1%
Exon 11/ Exon 11	15	2.6%
Exon 5/ Exon 5	15	2.6%
**Most frequent protein domains involved in variants#**		
Ring 0/ Ring 0	78	15.4%
Ubiquitin-like/ Ubiquitin-like	73	14.5%
Ring 0/ Ring 1	45	8.9%
Ubiquitin-like/ NA†	41	8.1%
Ubiquitin-like/ Ring 0	35	6.9%
Ring1/Ring1	32	6.3%

*There were 160 different combinations of exonic/ intronic (splice site variant) locations for the variants and therefore only the 8 most frequent locations have been listed.
# Similarly, there were 49 different combinations of protein domain locations for the variants and therefore only the most frequent locations have been listed. Of note 77 cases (13.2%) had 2 variants which couldn't be categorized into a specific protein domain.
† NA refers to cases where the second variant couldn't be categorized into a specific protein domain.

Supplementary figure 2: Flow chart demonstrating the number of patients included for each section of the analysis.

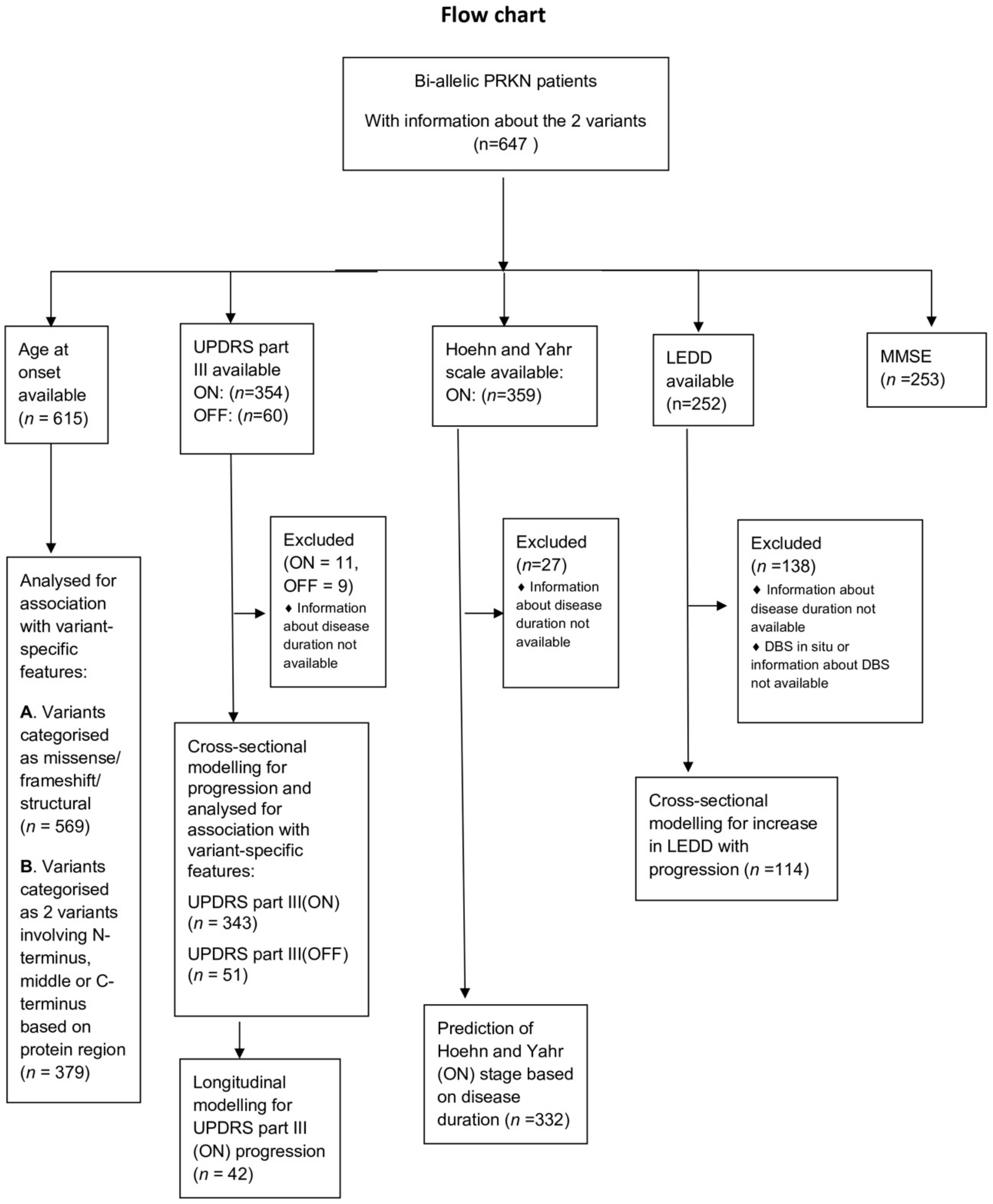

Supplementary figure 3: Boxplot demonstrating the average age at onset of *PRKN*-PD based on the type of variant and the sex of the individual.

(f/f = frameshift/frameshift, f/m = frameshift/ missense, f/s = frameshift/ structural, m/m = missense/ missense, m/s = missense/ structural, s/s = structural/ structural).

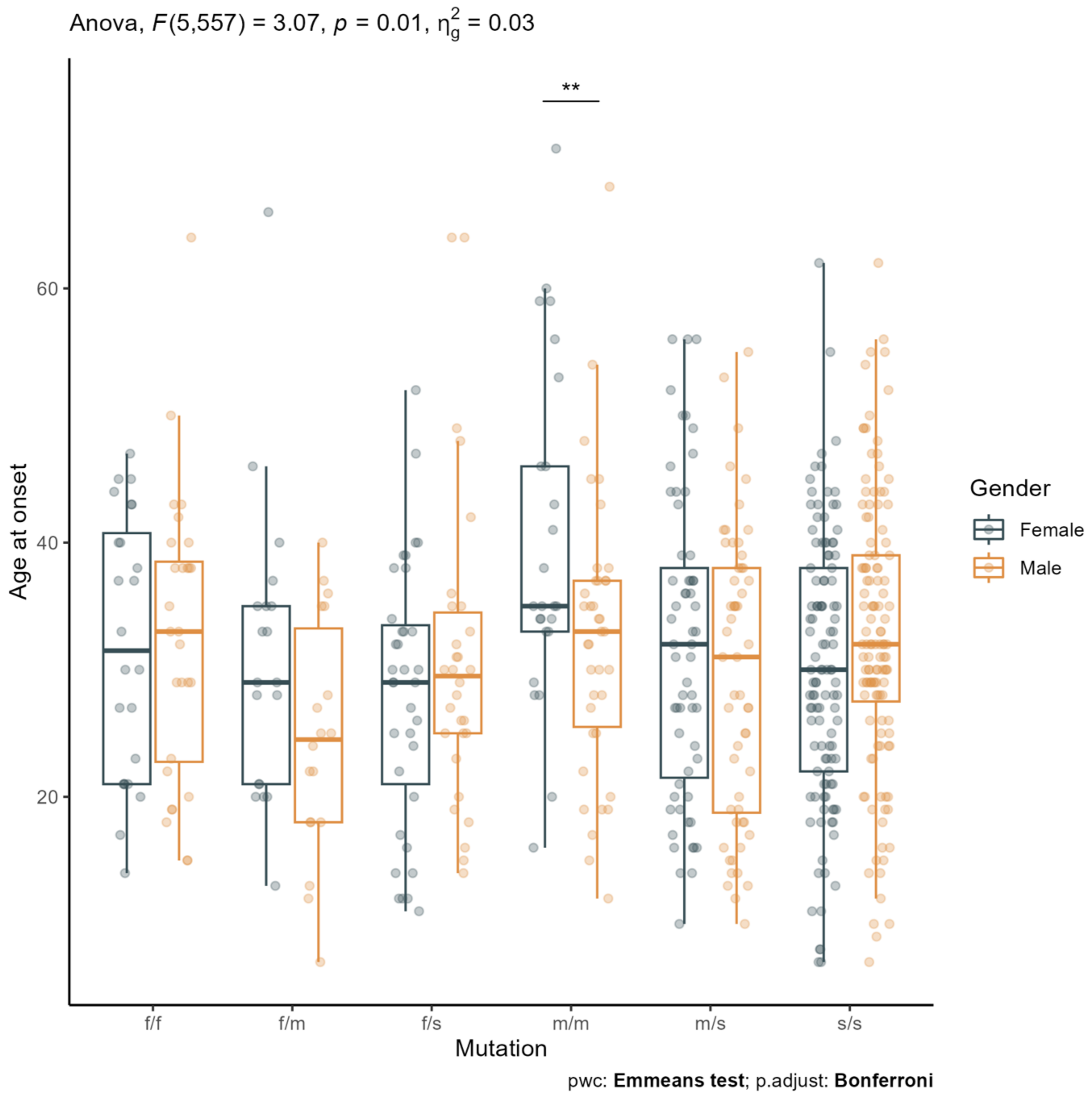

Supplementary figure 4: Histogram demonstrating age at onset of *PRKN*-PD in those with homozygous exon 3 deletions and homozygous N52Mfs*29 variants.

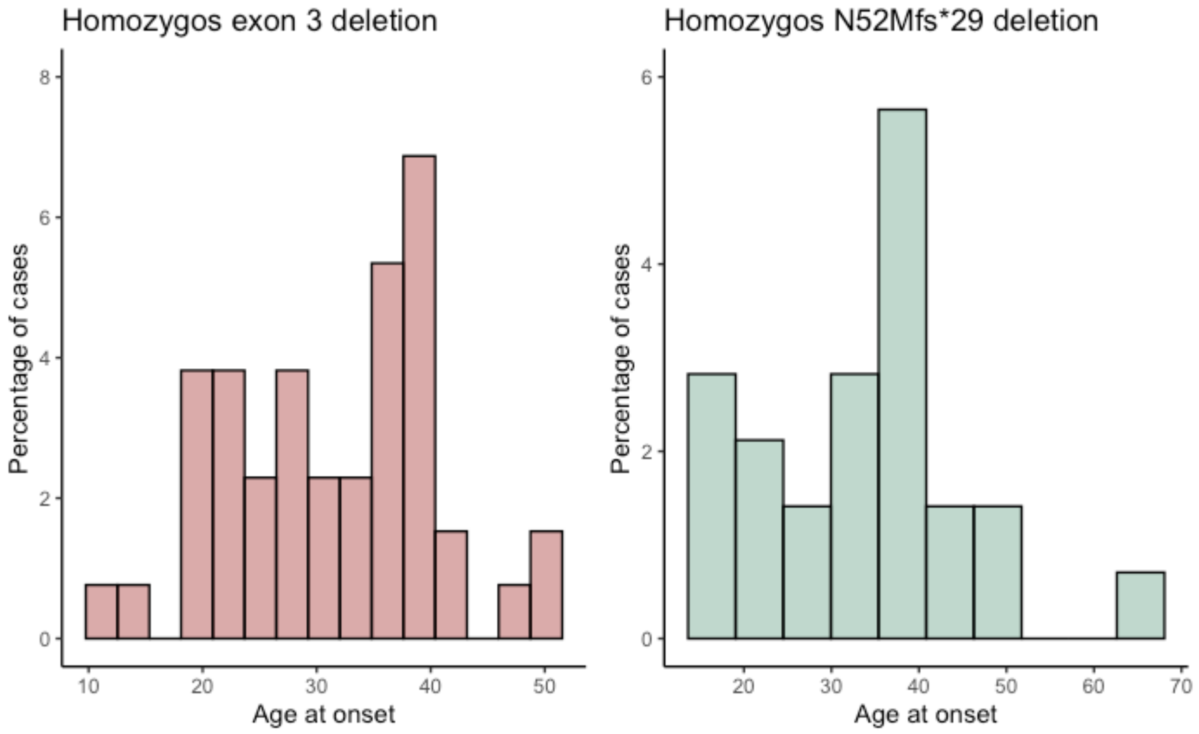

Supplementary figure 5: Flow chart demonstrating the number of patients included for analysis on detailed phenotypic features.

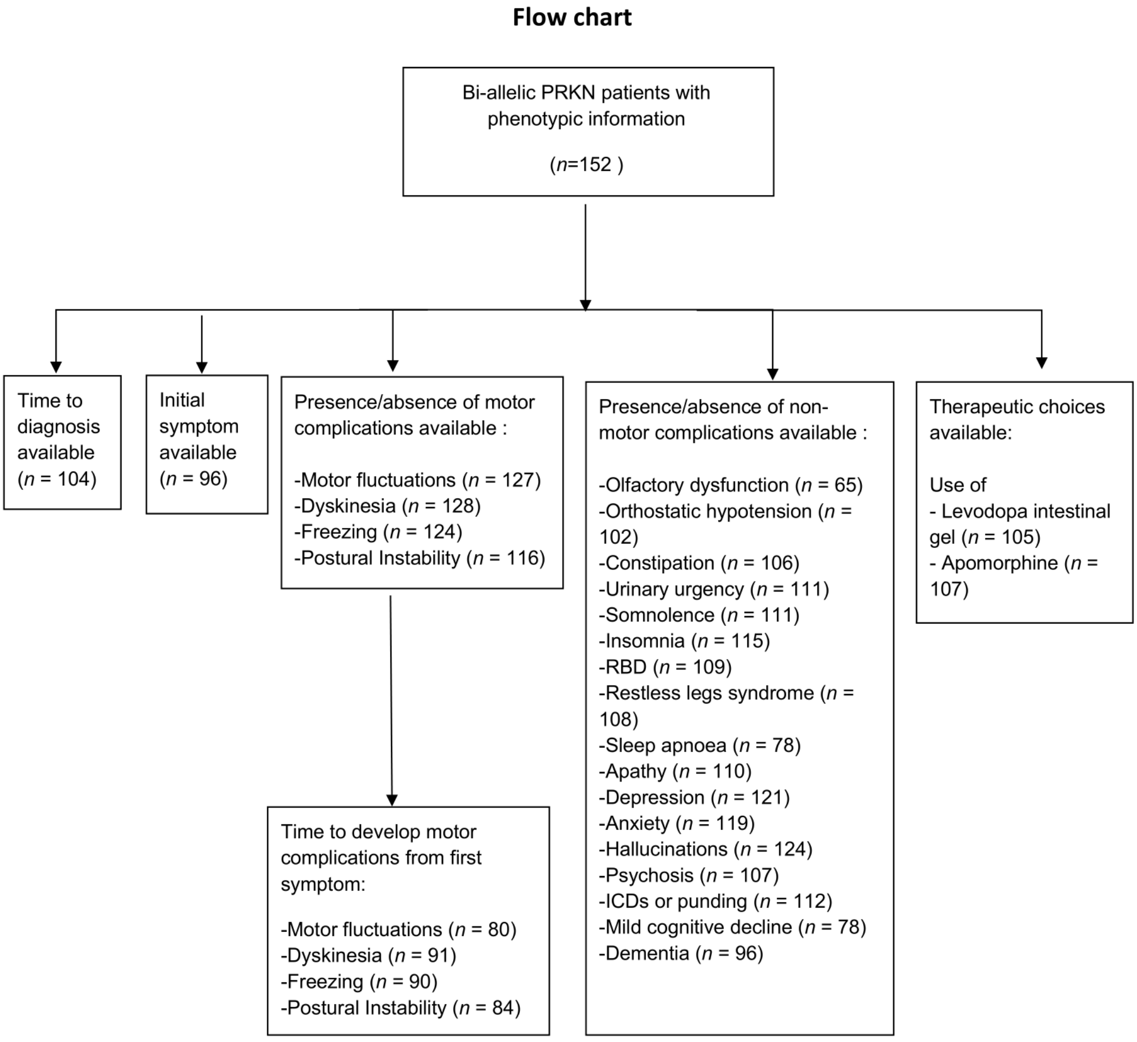

Supplementary figure 6: Linear regression model of UPDRS III (ON) progression adjusted for sex.

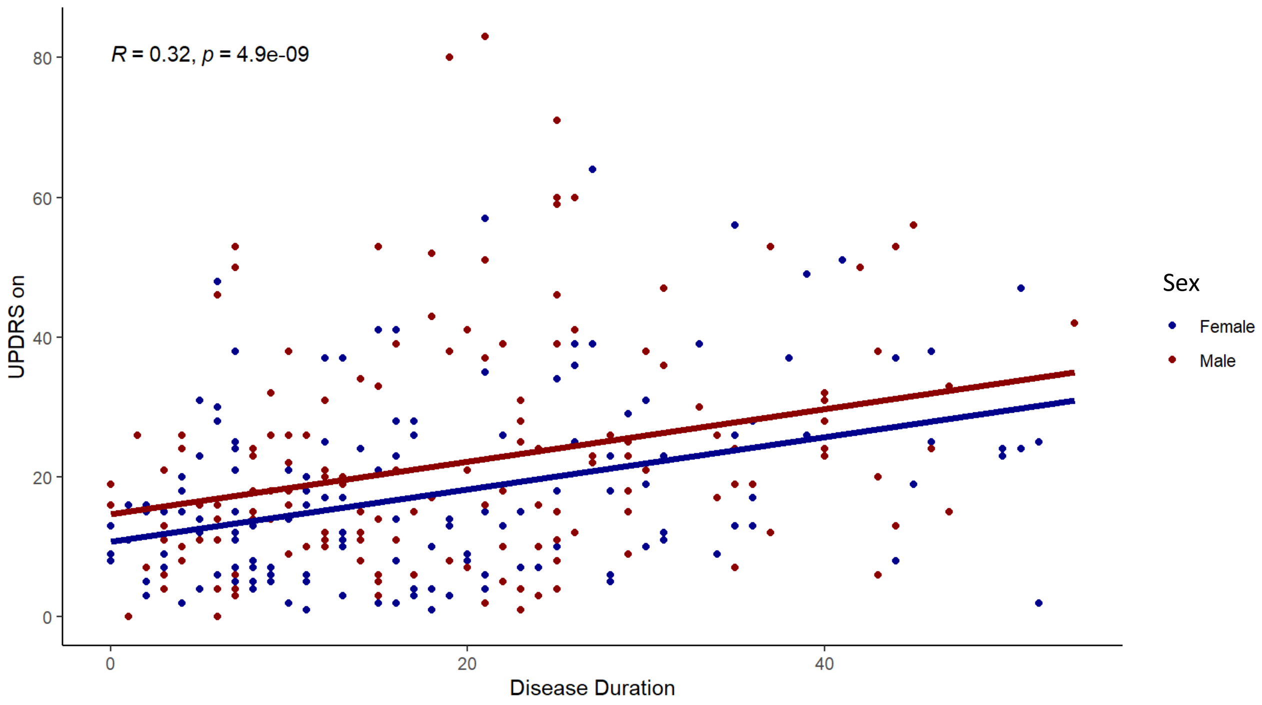

Supplementary table 5: Predicted probability of belonging to each Hoehn and Yahr stage based on disease duration.

Hoehn and Yahr Stage 0-1

Disease duration	Predicted	95% CI
0	0.52	[0.42, 0.60]
10	0.33	[0.27, 0.39]
20	0.19	[0.15, 0.23]
35	0.07	[0.04, 0.10]
55	0.02	[0.01, 0.03]

Hoehn and Yahr Stage 2

Disease duration	Predicted	95% CI
0	0.40	[0.33, 0.47]
10	0.50	[0.44, 0.56]
20	0.51	[0.45, 0.57]
35	0.35	[0.28, 0.42]
55	0.12	[0.06, 0.20]

Hoehn and Yahr Stage 3

Disease duration	Predicted	95% CI
0	0.07	[0.05, 0.11]
10	0.14	[0.11, 0.19]
20	0.25	[0.20, 0.30]
35	0.42	[0.34, 0.50]
55	0.40	[0.29, 0.52]

Hoehn and Yahr Stage 4

Disease duration	Predicted	95% CI
0	0.01	[0.01, 0.02]
10	0.03	[0.02, 0.05]
20	0.06	[0.04, 0.09]
35	0.16	[0.11, 0.23]
55	0.46	[0.31, 0.63]

Supplementary figure 7: Logistic regression model of probability of belonging to a Hoehn and Yahr stage based on disease duration.

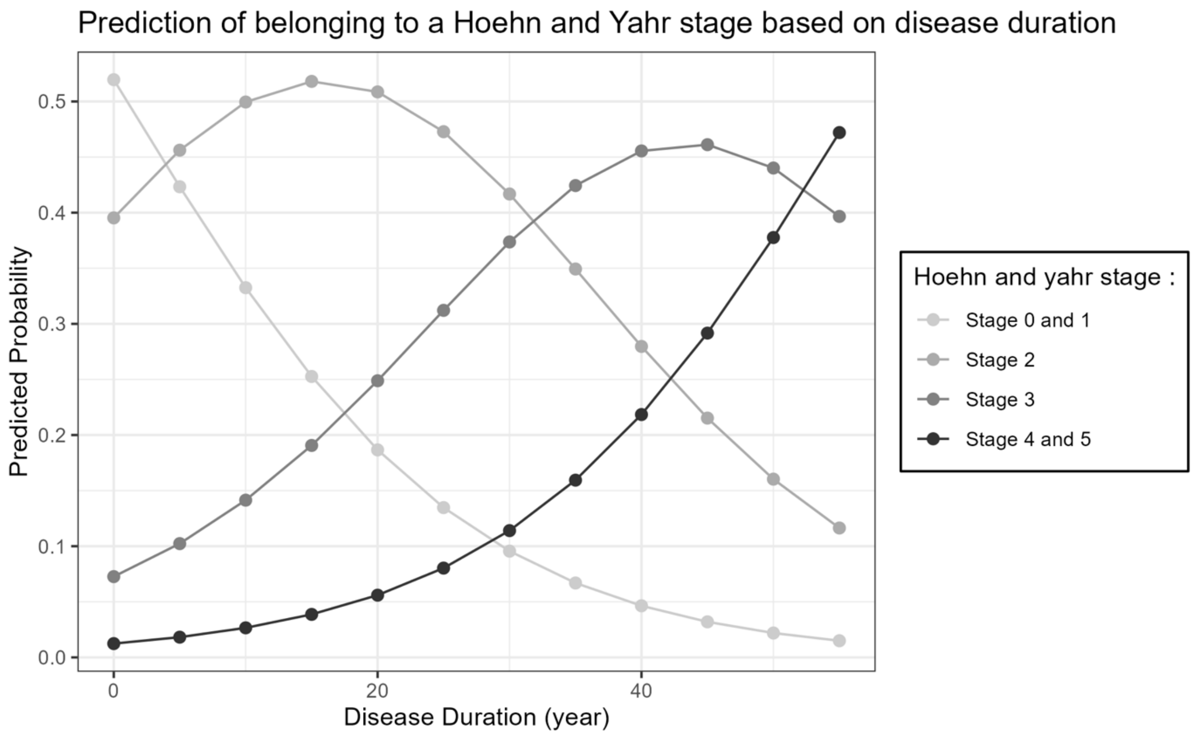

Supplementary figure 8: Boxplot comparing the age at onset of first symptom in the *PRKN*-PD cohort compared to the early-onset PD cohort

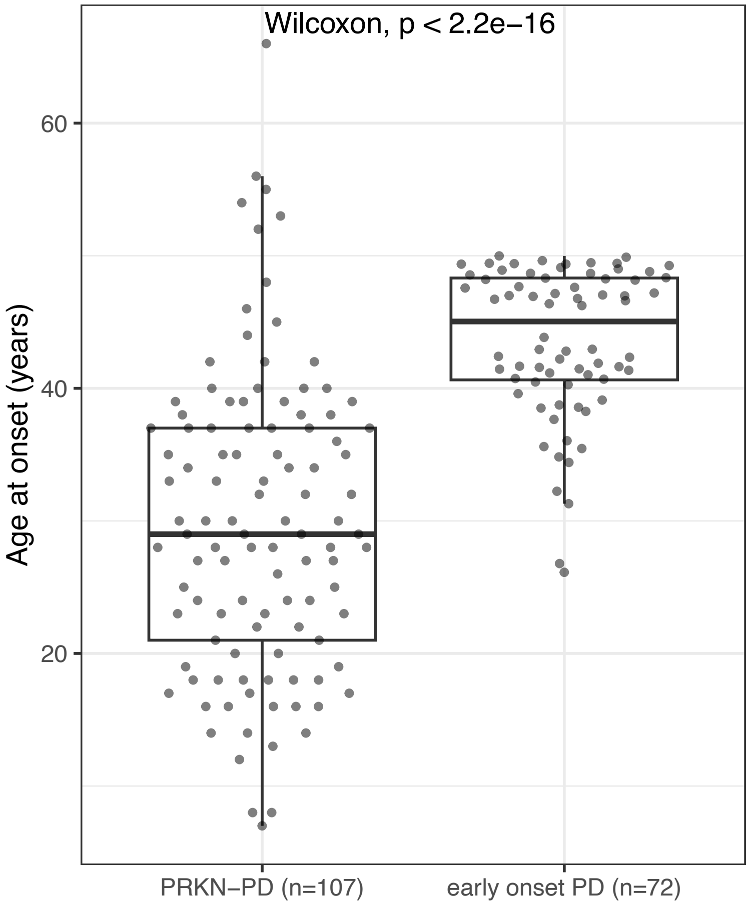

Supplementary table 6: List of centres that collaborated to the Genotype-Phenotype correlation in *PRKN*-PD (GPiP) study.

**GPiP centres**
1. Fondazione IRCCS Ca' Granda Ospedale Maggiore Policlinico, Milan, Italy
2. Institute of Neurogenetics, University of Lübeck, Lübeck, Germany
3. UCL Queen Square Institute of Neurology, University College London, London, United Kingdom
4. Department of Neurology, Hospital Universitari Mutua de Terrassa, and Fundació per a la Recerca Biomèdica i Social Mútua de Terrassa, Terrassa, Barcelona, Spain
5. National Institute of Health, Bethesda, United States
6. Hertie Institute for Clinical Brain Research, University of Tuebingen, Germany
7. Department of Neurology, MedUniVienna, Austria
8. The Dublin Neurological Institute at the Mater Misericordiae University Hospital, Dublin and University College Dublin, Ireland
9. Norwegian University of Science and Technology, Trondheim, Norway
10. The Neuro (Montreal Neurological Institute-Hospital), McGill University, Montreal, Quebec, Canada
11. Department of Neurology, University Hospitals Leuven, Belgium
12. University Clinical Center of Serbia, Neurology Clinic, Belgrade, Serbia

Appendix 1

The French clinicians' network for Parkinson's disease genetics (the PDG group) members: Yves Agid (site investigator, Department for the Central Nervous System, Paris), Mathieu Anheim (site investigator, Department of Neurology, Strasbourg), Michel Borg (site investigator, Department of Neurology, Nice), Alexis Brice (site investigator, Department of Genetics and Cytogenetics, Paris), Emmanuel Broussolle (site investigator, Pôle des Spécialités Neurologiques, Lyon), Jean-Christophe Corvol (site investigator, Center for Clinical Investigations, Paris), Philippe Damier (site investigator, Department of Neurology, Nantes), Luc Defebvre (site investigator, Service de Neurologie et Pathologie du Mouvement, Clinique Neurologique, Hôpital Roger Salengro, Lille), Alexandra Dürr (site investigator, Department of Genetics and Cytogenetics, Paris), Franck Durif (site investigator, Department of Neurology A, Clermont-Ferrand), Jean Luc Houetto (site investigator, service de neurologie, CHU de Poitiers, Poitiers), Paul Krack (site investigator, Pôle Psychiatrie et Neurologie, Grenoble), Stephan Klebe (site investigator, Centre for Clinical Investigations, Paris), Suzanne Lesage (site investigator, ICM INSERM U1127, Paris), Ebba Lohmann (site investigator, Department of Genetics and Cytogenetics, Paris), Maria Martinez (site investigator, INSERM Unit 563, Toulouse), Graziella Mangone (site investigator, Centre for Clinical Investigations, Paris), Pierre Pollak (site investigator, Pôle Psychiatrie et Neurologie, Grenoble), Olivier Rascol (site investigator, Clinical Investigation Centre, Toulouse), François Tison (site investigator, Pôle des Neurosciences, Cliniques de Neurologie, Bordeaux), Christine Tranchant (site investigator, Department of Neurology, Strasbourg), Marc Vérin (site investigator, Department of Neurology, Rennes), François Viallet (site investigator, Department of Neurology, Aix-en-Provence), and Marie Vidailhet (site investigator, Department of Neurology, Paris).
